# Tubulin‐Based Microtentacles Aid in Heterotypic Clustering of Neutrophil‐Differentiated HL‐60 Cells and Breast Tumor Cells

**DOI:** 10.1002/advs.202409260

**Published:** 2024-12-18

**Authors:** Julia A. Ju, Keyata N. Thompson, David A. Annis, Makenzy L. Mull, Darin E. Gilchrist, Aidan Moriarty, Katarina T. Chang, Megan B. Stemberger, Michael J. Noto, Michele I. Vitolo, Stuart S. Martin

**Affiliations:** ^1^ Marlene and Stewart Greenebaum NCI Comprehensive Cancer Center University of Maryland School of Medicine 655 W. Baltimore St. Baltimore MD 21201 USA; ^2^ Graduate Program in Molecular Medicine University of Maryland School of Medicine 800 W. Baltimore St. Baltimore MD 21201 USA; ^3^ Graduate Program in Epidemiology and Human Genetics University of Maryland Baltimore 800 W. Baltimore St. Baltimore MD 21201 USA; ^4^ Division of Pulmonary, Critical Care, and Sleep Medicine Department of Medicine University of Maryland School of Medicine 22 S. Greene St. Baltimore MD 21201 USA; ^5^ Department of Pharmacology and Physiology University of Maryland School of Medicine 655 W. Baltimore St. Baltimore MD 21201 USA; ^6^ United States Department of Veterans Affairs VA Maryland Health Care System Baltimore MD 21201 USA

**Keywords:** circulating tumor cells, heterotypic cluster, HL‐60 cells, metastasis, microtentacles, neutrophils, TetherChip

## Abstract

Circulating tumor cells (CTCs) travel through the vasculature to seed secondary sites and serve as direct precursors of metastatic outgrowth for many solid tumors. Heterotypic cell clusters form between CTCs and white blood cells (WBCs) and recent studies report that a majority of these WBCs are neutrophils in patient and mouse models. The lab discovered that CTCs produce tubulin‐based protrusions, microtentacles (McTNs), which promote reattachment, retention in distant sites during metastasis and formation of tumor cell clusters. Neutrophil‐CTC clusters help CTCs survive the harsh vascular environment to promote successful metastasis, however, the specific mechanism of this interaction is not fully understood. Utilizing TetherChip technology, it is found that primary and differentiated neutrophils produce McTNs composed of detyrosinated and acetylated α‐tubulin and vimentin. Neutrophil McTNs aid in cluster formation, migration, and reattachment, which are suppressed with the tubulin‐depolymerizing agent, Vinorelbine. Co‐culturing differentiated neutrophils and tumor cells formed heterotypic clusters that enhanced migration. CTC‐neutrophil clusters have higher metastatic efficiency, and by demonstrating that neutrophils form McTNs, a new possible mechanism for how neutrophils interact with tumor cells is revealed. These findings further support the idea that developing cluster‐disrupting therapies can provide a new targeted strategy to reduce the metastatic potential of cancer cells.

## Graphical abstract

Utilizing TetherChip technology, the authors revealed that primary and differentiated neutrophils produce tubulin‐based microtentacles. These microtentacles facilitated homotypic clustering, reattachment, and heterotypic clustering, which are inhibited with the tubulin depolymerizer, Vinorelbine. Co‐culturing neutrophils and tumor cells enhanced migration and allowed migration toward multiple stimuli. Taken together, these results illustrate a new potential mechanism for interactions between tumor cells and neutrophils.

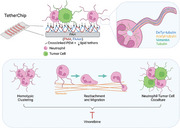

## 1 Introduction

Currently, the majority of treatments for cancer patients rely on targeting the growth of the primary tumor rather than metastasis. However, metastasis remains the main cause of treatment resistance and patient mortality, which is why the need for metastasis‐specific therapies are essential for improving patient outcome.^[^ ^1^ ^]^ This is challenging though, because cells that constitute these secondary metastatic sites are often molecularly distinct from the primary tumor. Circulating tumor cells (CTCs) are considered direct precursors of metastasis and are defined as cells that have shed from the primary tumor and traverse the bloodstream to seed at secondary distant sites.^[^ ^2^ ^]^ Before tumor cells are able to colonize at distant metastatic sites, however, they encounter and engage with a variety of immune cells or white blood cells (WBCs) throughout the bloodstream, which ultimately can regulate tumor progression.

Neutrophils represent a majority of the circulating leukocyte cell population in human blood and are most often known for their role in human innate immunity under normal physiological conditions.^[^ ^3^ ^]^ They are produced in the bone marrow, circulate through the bloodstream, migrate to distant tissues to carry out their functions, and are eventually eliminated by macrophages.^[^ ^4^ ^]^ Neutrophils are fundamental in inflammatory responses and eliminate pathogens by phagocytosis, degranulation, cytokine production, the release of reactive oxygen species, or by releasing neutrophil extracellular traps (NETS), which are crucial for clearance of an infection.^[^ ^5^ ^]^ Over the last decade, however, the role of neutrophils has been greatly expanded beyond just innate immunity and acute inflammation with key functions in cancer being identified as well. In contrast to their physiological roles to activate the immune system as a defense mechanism, it has recently become increasingly apparent that neutrophils can also play a tumor‐supportive and immunosuppressive function in the context of tumor progression.^[^ ^6^, ^7^, ^8^ ^]^ Increases in the circulating neutrophil‐to‐lymphocyte ratio has become an indicator of poor overall survival and has been used as a prognostic marker in many cancer types.^[^ ^9^ ^]^ Additionally, circulating neutrophils were found to promote CTC survival by suppressing peripheral leukocyte activation to enhance metastasis.^[^ ^10^ ^]^

It has been well‐established that CTCs can be found within the bloodstream as single CTCs or as either homotypic or heterotypic CTC clusters.^[^ ^11^ ^]^ Homotypic clusters are composed of only cancer cells whereas heterotypic clusters can contain CTCs and immune cells, stromal cells, or platelets. Studies have shown that CTCs which form either homotypic or heterotypic clusters have a 50–100x higher metastatic potential and are associated with worse prognosis and decreased overall survival in patients compared to single CTCs.^[^ ^7^, ^11^, ^12^ ^]^ It was recently demonstrated that tumor cells form heterotypic clusters with neutrophils via vascular cell adhesion molecule ‐1 (VCAM‐1) and that this direct association gives CTCs a proliferative survival advantage to enhance metastatic potential.^[^ ^7^ ^]^ Intracellular adhesion molecule ‐1 (ICAM‐1) and macrophage‐1 antigen (Mac‐1) were also shown to facilitate the interaction between circulating neutrophils and CTCs to promote liver metastasis.^[^ ^13^ ^]^ Lipopolysaccharide (LPS)‐stimulated neutrophils formed heterotypic aggregates with tumor cells in zebrafish models, which enhanced the extravasation potential of tumor cells.^[^ ^6^ ^]^ Given these findings, it was proposed that neutrophils could cluster and surround tumor cells to protect them from immune cell attacks while also helping transport them through the bloodstream to a metastatic tissue site,^[^ ^14^ ^]^ however, this hypothesis and the exact mechanisms of this model are still being investigated.

During metastasis, tumor cells circulate throughout the non‐adherent environments of the bloodstream and lymphatics. Our lab discovered that under these non‐adherent conditions, epithelial tumor cells form unique microtubule‐based protrusions, called microtentacles (McTNs), that result from an imbalance of physical forces between the actin cortex and microtubule network.^[^ ^15^ ^]^ McTNs are composed of both vimentin intermediate filaments and post‐translationally modified forms of α‐tubulin, including acetylated and detyrosinated α‐tubulin.^[^ ^16^, ^17^, ^18^ ^]^ Both of these modifications are associated with a more invasive phenotype and are poor prognostic indicators in breast cancer patients.^[^ ^19^, ^20^, ^21^, ^22^ ^]^ We have shown that McTNs on tumor cells aid in reattachment to endothelial cells,^[^ ^16^ ^]^ promote homotypic tumor cell clustering^[^ ^23^ ^]^ and positively correlate with tumor cell aggressiveness and enhanced metastatic potential in four different genetic models.^[^ ^24^, ^25^, ^26^, ^27^, ^28^ ^]^ McTNs are formed from the growing end of the αβ tubulin dimer only when a cell is in a suspended or free‐floating state. Therefore, to overcome the technical challenges of imaging McTNs and non‐adherent cells, our lab developed a microfluidic platform, TetherChip, that recapitulates the free‐floating environment by preventing cell‐ adhesion with an optically‐clear polyelectrolyte multilayer (PEM) nanosurface combined with a terminal lipid anchor to immobilize suspended cells in place (Figure 2G).^[^ ^29^, ^30^ ^]^ With a partial thermal imidization crosslinking reaction of the PEM deposited onto microfluidic slides, we are able to analyze several metastatic phenotypes such as McTN production,^[^ ^29^ ^]^ clustering efficiency,^[^ ^23^ ^]^ sphere formation,^[^ ^31^ ^]^ and drug response.^[^ ^23^ ^]^ We previously demonstrated that TetherChip allowed the comparison of multiple epithelial tumor cell types, revealing that McTNs are not unique to breast cancer cells.^[^ ^29^, ^32^ ^]^ Importantly, these tumor types that were shown to produce McTNs (lung, colon, breast, prostate, ovarian) already represent over 50% of all human solid tumors. This is significant because epithelial carcinomas represent 90% of human solid tumors.^[^ ^33^ ^]^ By directly integrating TetherChip into CTC isolation devices for efficient capture of CTCs, we expanded its applications to successfully process clinical liquid biopsy samples in a streamlined workflow.^[^ ^29^ ^]^ Using TetherChip, this study aimed to explore whether neutrophil‐tumor cell clustering could be mediated by McTNs and if this association could ultimately be inhibited using an already FDA‐approved anti‐microtubule therapy to reduce the metastatic potential of heterotypic clusters.

Despite the growing interest in understanding the mechanisms of neutrophil‐CTC clustering in metastasis, many technical challenges remain due to their short lifespan. Mature neutrophils are terminally differentiated, do not proliferate, and begin to undergo apoptosis within 24 h in the bloodstream and within 12 h of isolation from the blood,^[^ ^5^ ^]^ limiting the feasibility of longer‐term and genetic manipulation experiments. To overcome this, human myeloid cell line model systems, using either HL‐60, PLB985, or NB4 cells, have been developed and their ability to undergo neutrophil‐like differentiation in vitro has been thoroughly characterized.^[^ ^34^, ^35^ ^]^ These neutrophil‐like cells have been used as a model to study various neutrophil functions including phagocytosis, chemotaxis, oxidative burst, NETosis, and degranulation.^[^ ^36^ ^]^ HL‐60 cells are a promyelocytic leukemia cell line that are most commonly used for neutrophils studies and can be differentiated into neutrophils via the addition of all‐trans retinoic acid (ATRA) or polar‐planar compounds (dimethyl sulfoxide (DMSO) and dimethylformamide (DMF)) and less frequently with the use of actinomycin D or dibutyryl cyclic AMP (dbcAMP).^[^ ^37^ ^]^ ATRA is thought to induce differentiation by regulating transcription through the retinoic acid receptor (RARα) and dbcAMP through cAMP signaling, though the mechanisms of DMSO‐induced neutrophil differentiation are not as clear.^[^ ^38^ ^]^ HL‐60 cells can also be differentiated into monocyte‐like cells using Vitamin D_3_ or macrophage‐like cells using phorbol esters (TPA).^[^ ^39^ ^]^ However, it is well‐established that DMSO‐based differentiation results in primarily a neutrophil‐like phenotype and is also the most efficient at inducing neutrophil‐specific surface markers on HL‐60 cells while maintaining the highest degree of cell viability compared to treating with ATRA, DMF, or dbcAMP.^[^ ^38^ ^]^ Because of this, we decided to conduct the majority of our experiments using DMSO‐differentiated HL‐60 cells to study neutrophils and how they may interact with tumor cells.

In this study, we show for the first time that both primary neutrophils and neutrophils differentiated from HL‐60 cells form tubulin‐based McTNs that were composed of detyrosinated α‐tubulin, acetylated α‐tubulin, and vimentin, similar to studies previously described in epithelial tumor cells. Differentiation into neutrophils increased McTN‐mediated phenotypes including cell reattachment and homotypic clustering, which could be inhibited with an FDA‐approved tubulin‐depolymerizing agent, Vinorelbine. Differentiated neutrophils and tumor cells formed heterotypic clusters that could also be disrupted with Vinorelbine. Finally, we illustrate that co‐culturing differentiated neutrophils and tumor cells enhanced migration compared to each cell type individually and gave the heterotypic clusters the capacity to migrate toward multiple stimuli. Overall, our results prove for the first time that immune cells such as neutrophils can form McTNs and that these McTNs facilitate reattachment to extracellular matrix (ECM), homotypic and heterotypic clustering, and that the heterotypic cluster promotes enhanced migration.

## 2 Results

### 2.1 Primary Neutrophils Produce Tubulin‐Based Microtentacles

**Figure 1:**
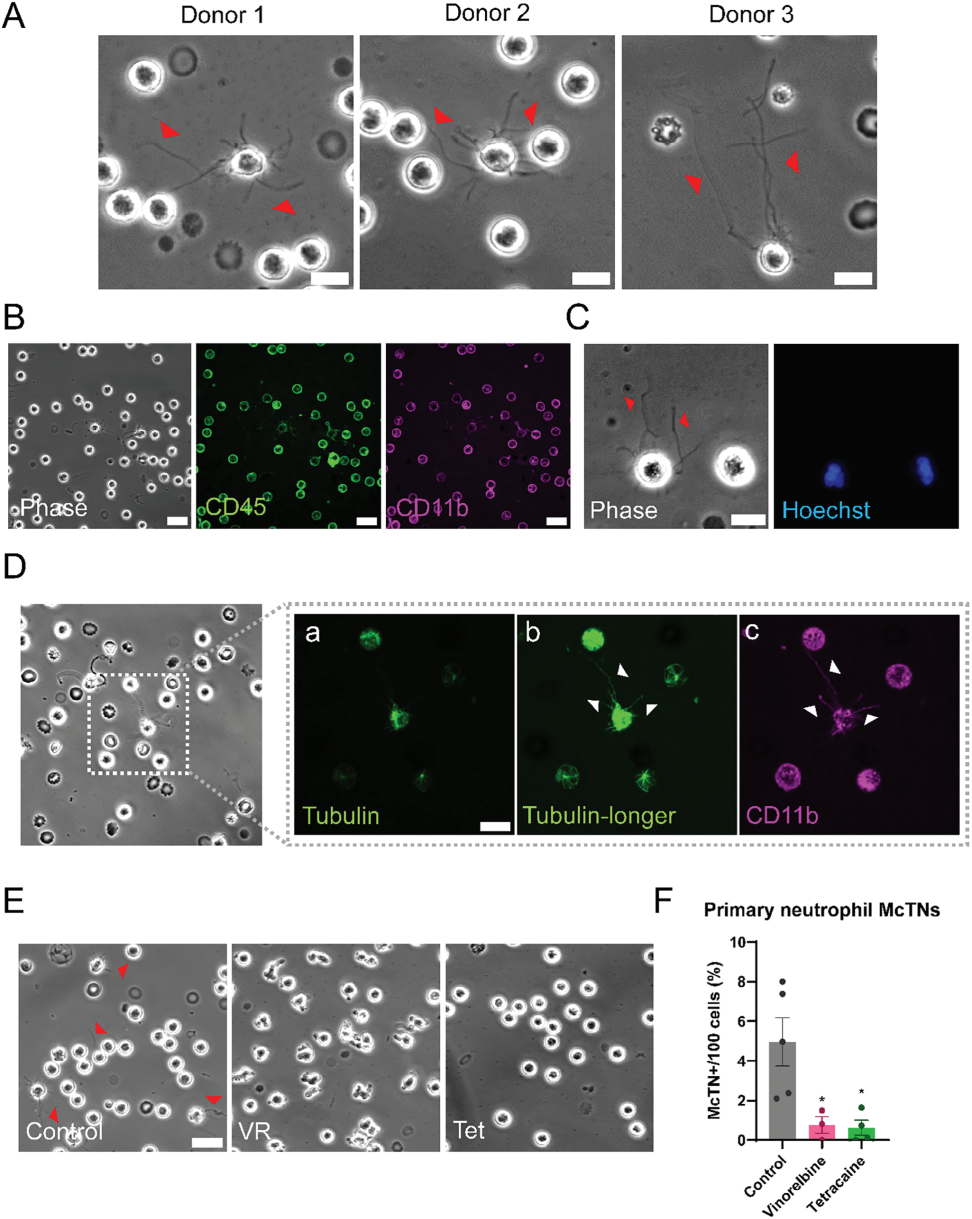
Primary human neutrophils produce tubulin‐based McTNs. A) Representative phase contrast images of live isolated primary human neutrophils from three separate human donors. Red arrows indicate the presence of McTNs. Scale bar = 10 µm. B) Representative phase contrast (left), CD45‐stained (middle) and CD11b‐stained (right) live primary neutrophils illustrating the cells that were isolated are neutrophils. Scale bar = 20 µm. C) Representative phase contrast (left) and Hoechst‐stained (right) live primary neutrophils displaying that McTNs are not made of DNA. Red arrows indicate microtentacles. Scale bar = 10 µm. D) Representative phase contrast image of isolated live primary human neutrophils with McTNs stained with a) a live tubulin‐tracker, b) the same image as (a) with the majority of the cell necessarily overexposed to reveal the thin, fluorescently labeled McTNs and c) CD11b. Scale bar = 10 µm. E) Representative phase contrast images of control neutrophils (left) or neutrophils treated with 10 µM Vinorelbine (VR, middle) or 250 µM Tetracaine (Tet, right) for 1 hr on TetherChips. Red arrows indicate McTNs. Scale bar = 20 µm. F) McTN quantification of live neutrophils tethered onto TetherChips and treated for 1 hr. Error bars indicate mean ± standard error of mean, n = 3–5 with at least 100 cells counted per condition per replicate. *p < 0.05 versus control (One‐way ANOVA with Bonferroni post‐ test).

It has been shown that neutrophil‐CTC heterotypic clusters form in the bloodstream and these clusters are correlated to a reduced progression‐free survival compared to both single CTCs and homotypic CTC clusters in patients.^[^ ^7^ ^]^ Our lab has also shown that McTNs aid in homotypic tumor cell clustering.^[^ ^15^, ^23^ ^]^ To determine whether neutrophils also produced McTNs that could potentially facilitate heterotypic clustering, primary neutrophils were isolated from the blood of three different healthy human donors and tethered onto TetherChips (**Figure** **1**A; Figure S1A, Supporting Information, **Figure** **2**G). A small percentage of neutrophils from all three donors produced long and dynamic protrusions (Figure 1A, red arrows) (quantified in Figure 1F, control). Staining with both CD45 and CD11b confirmed our freshly isolated cells to be neutrophils (Figure 1B; Figure S1B, Supporting Information), and the Hoechst staining (Figure 1C; Figure S1C, Supporting Information) confirmed the neutrophils were healthy, thus the protrusions (red arrows) were not NETs containing DNA nor a consequence of cell death. Since our lab has previously demonstrated that McTNs are tubulin‐based, the neutrophils were stained with a live tubulin tracker (Figure 1D panels a and b) as well as CD11b (Figure 1D panel c) to illustrate that tubulin is present throughout the protrusions and confirm the neutrophil status. To verify that these protrusions were tubulin‐driven McTNs, the neutrophils were treated with Vinorelbine (VR), a microtubule depolymerizer, as well as Tetracaine (Tet), a kinesin inhibitor (Figure 1E). Quantification of McTNs displayed that both treatments significantly reduced protrusion frequency compared to the control neutrophils (Figure 1F) illustrating the protrusions are inhibited by tubulin‐modulating drugs further supporting they are tubulin‐based McTNs. These results confirm that a small percentage of primary neutrophils have tubulin‐based McTNs. As neutrophils start to undergo apoptosis after 12 h of being isolated from the blood and cannot be cultured long‐term, neutrophils differentiated from HL‐60 cells via 1.3% DMSO were used for the remaining experiments.

**Figure 2:**
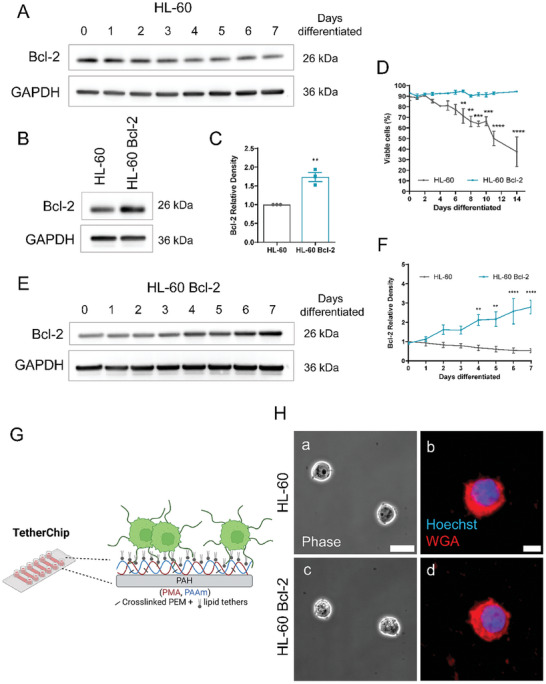
Bcl‐2 overexpression in HL‐60 cells prevents apoptosis upon differentiation into neutrophils. A) Protein levels of Bcl‐2 in HL‐60 cells that were differentiated over 7 days were measured via Western blotting analysis. B) Immunoblot of Bcl‐2 in HL‐60 cells or HL‐60 cells stably overexpressing Bcl‐2. C) Densitometric fold change analysis of Bcl‐2 protein expression normalized to GAPDH and fold change over the parental HL‐60 cells. Error bars indicate ± standard error of mean, n = 3. **p < 0.01 versus HL‐60 (Two‐tailed t‐test). D) Cell viability of HL‐60 and HL‐60 Bcl‐2 cells was determined using a trypan blue exclusion assay using an automated cell counter at 0, 1, 2, 3, 4, 5, 6, 7, 8, 9, 10, 11 and 14 days post‐differentiation with 1.3% DMSO. Error bars indicate ± standard error of mean, n = 3. **p < 0.01; ***p < 0.001; ****p < 0.0001 versus HL‐60 (Two‐way ANOVA with Bonferroni post‐test). E) Protein levels of Bcl‐2 in HL‐60 Bcl‐2 overexpressing cells that were differentiated over 7 days were measured via Western blotting analysis. F) Densitometric fold change analysis of Bcl‐2 protein expression from western blot analysis in A and E normalized to GAPDH. Error bars indicate standard error of mean, n = 3–5. **p < 0.01, ****p < 0.0001 versus HL‐60 at the indicated time point (Two‐way ANOVA with Bonferroni post‐test). G). Diagram of TetherChip illustrating how cells can be immobilized by the lipid tether while also not adhering to the surface via the polyelectrolyte multilayers. H‐a,c) Phase contrast images of undifferentiated HL‐60 cells and HL‐60 Bcl‐2 cells, respectively. Images were taken at 60x magnification on a Nikon Ti2‐E inverted microscope. Scale bar = 10 µm. b,d) Immunofluorescent images of HL‐60 and HL‐60 Bcl‐2 cells stained with Hoechst and Wheat Germ Agglutinin AlexaFluor 594 (WGA) taken at 60x magnification using an Olympus IX81 microscope with a Fluoview FV1000 confocal laser scanning system. Scale bar = 5 µm. From here on out only HL‐60 Bcl‐2 cells were used for experiments.

### 2.2 Bcl‐2 Overexpression in HL‐60 Cells Prevents Differentiation‐Induced Apoptosis

Previous studies have shown that differentiation of HL‐60 cells into neutrophils by retinoic acid, phorbol 12‐myristate 13‐acetate (PMA), and DMSO led to a gradual increase in cell death over time by activation of an apoptotic pathway.^[^ ^40^, ^41^ ^]^ To verify our cells behaved similarly to what the literature described, HL‐60s were differentiated with 1.3% DMSO over the course of 7 days and B‐cell lymphoma‐2 (Bcl‐2), protein expression was measured (Figure 2A,F). As expected, incubation of HL‐60s with DMSO resulted in a progressive decrease in endogenous Bcl‐2 with day 7 resulting in the cells expressing ≈50% Bcl‐2 levels compared to Day 0 (Figure 2F, gray line). Overexpressing Bcl‐2 in HL‐60 cells can protect them from decreased cell viability and also has no significant effects on the differentiation process itself.^[^ ^42^ ^]^ We decided that introducing Bcl‐2 overexpression into our HL‐60 cells would be advantageous to downstream functional assays that would require us to use terminally differentiated cells for several days without a loss in cell viability. HL‐60 cells were then transduced with a Bcl‐2‐containing lentiviral vector for all further experiments and basal Bcl‐2 protein levels were quantified in order to verify moderate overexpression (Figure 2B,C). To confirm that Bcl‐2 overexpression prevented cell death, both normal HL‐60s and HL‐60 Bcl‐2 cells were differentiated for two weeks, and viability was assessed using a trypan blue exclusion assay (Figure 2D). Overexpression of Bcl‐2 significantly increased viability after 7 days of differentiation with 94% of the cells remaining alive after 14 days of differentiation. On the other hand, the normal HL‐60 cells without Bcl‐2 overexpression declined to ≈38% viable after 14 days of differentiation. This gave us at least a one‐week time frame with the Bcl‐2 overexpressed cells after complete differentiation into neutrophils (7 days) to perform longer‐term functional assays that would not have been possible with the normal HL‐60s. HL‐60 Bcl‐2 cells were then differentiated over 7 days and Bcl‐2 protein expression was quantified (Figure 2E). After 7 days, these cells maintained Bcl‐2 expression and even had increased expression by 3‐fold (Figure 2F teal line). Representative images of both undifferentiated parental HL‐60 cells (Figure 2H‐a,b) and HL‐60 Bcl‐2 cells (Figure 2H‐c,d) illustrate there are no significant morphology differences between them in both phase contrast (panels a and c) and when tethered on TetherChip (Figure 2G) and stained fluorescently with wheat germ agglutinin (WGA, plasma membrane stain, panels b and d). These results confirmed that Bcl‐2 protected HL‐60 cells from differentiation‐induced apoptosis while providing a platform for us to study neutrophils without having difficulties associated with cell viability in the process. All subsequent experiments in this study were performed using the HL‐60 Bcl‐2 cells differentiated into neutrophils.

### 2.3 Differentiation into Neutrophils Induces Microtentacle Formation

**Figure 3:**
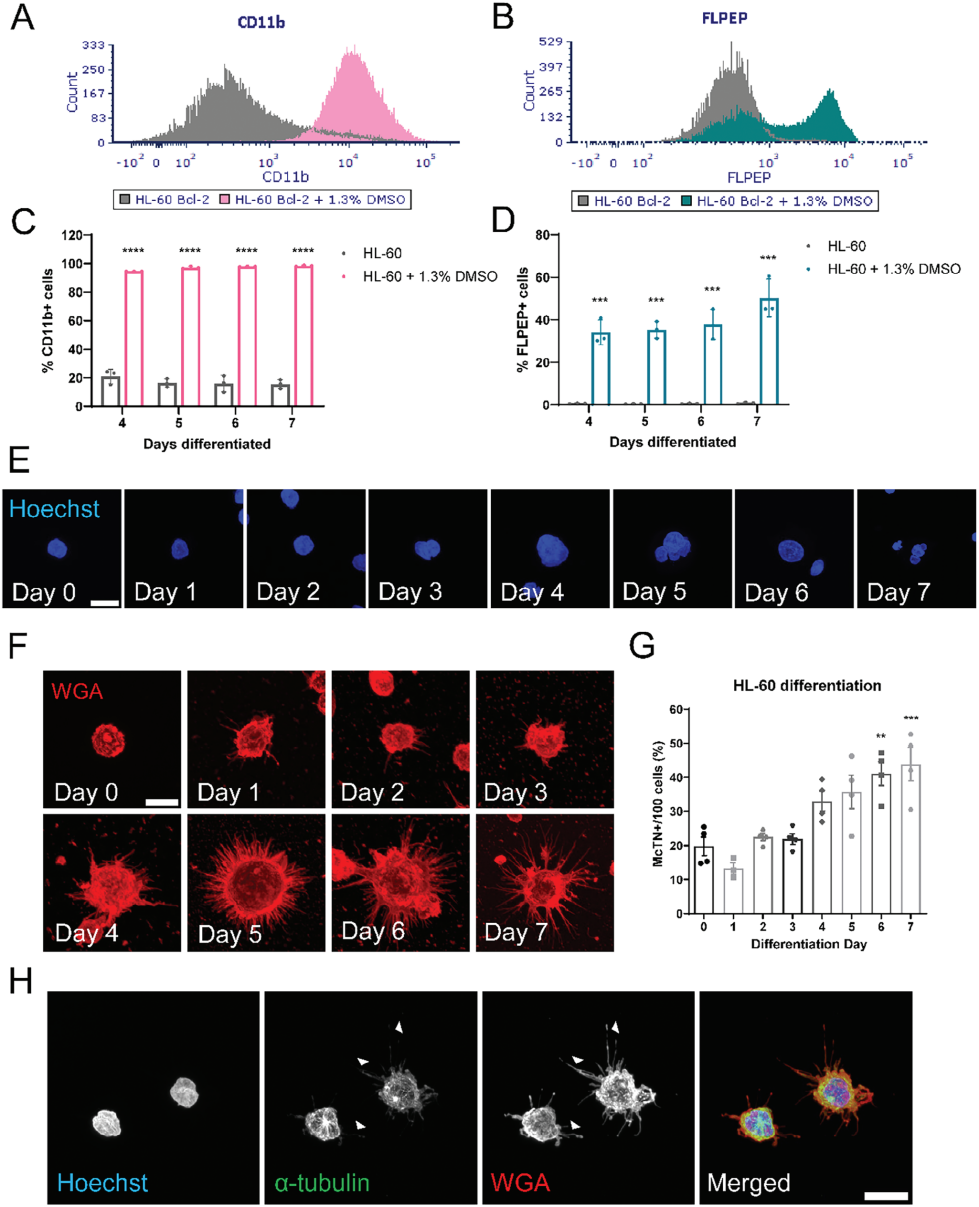
Differentiation of HL‐60 cells into neutrophils induces McTN formation. HL‐60 Bcl‐2 cells were differentiated into a neutrophil‐like state by culturing in 1.3% DMSO media for 7 days. A) Undifferentiated and differentiated cells were stained with an antibody against CD11b, chosen as an early differentiation marker and with B) FLPEP (a fluorescent ligand of FPR1), chosen as a late differentiation marker. Samples were measured by flow cytometry and data was analyzed using FCS Express software. C) Averaged values of CD11b positive cells between 4 and 7 days of differentiation. Error bars indicate mean ± standard deviation, n = 3. ****p < 0.0001 versus the undifferentiated sample for each time point (Two‐tailed t‐test). D) Averaged values of FLPEP positive cells between 4 and 7 days of differentiation. Error bars indicate mean ± standard deviation, n = 3. ***p < 0.001 versus the undifferentiated sample for each time point (Two‐tailed t‐test). E) Immunofluorescent images of tethered and fixed HL‐60 Bcl‐2 cells differentiated over the course of 7 days stained with Hoechst and F). WGA. Images were taken at 60x magnification using an Olympus IX81 microscope with a Fluoview FV1000 confocal laser scanning system. Scale bar = 10 µm. G) McTN quantification of tethered and fixed HL‐60 Bcl‐2 cells differentiated over 7 days. Data represents quantification of McTN frequency from four independent experiments with 100 cells counted for each. Error bars indicate ± standard error of mean, n = 4. **p < 0.01; ***p < 0.001 versus day 0 (One‐way ANOVA with Bonferroni post‐test). H) Immunofluorescent images of tethered and fixed day 7 differentiated neutrophils stained with Hoechst, α‐tubulin, and WGA were taken at 60x magnification using a Nikon Ti2‐E inverted microscope with a Nikon AX‐R confocal system. Images were denoised in a post‐processing step using NIS Elements. Scale bar = 10 µm.

To verify successful differentiation, both early and late differentiation markers were measured with flow cytometry (Figures S2 and S3, Supporting Information) and by RNA‐Sequencing (RNASeq) analysis (Figure S4, Supporting Information) in undifferentiated HL‐60 Bcl‐2 cells and differentiated neutrophils. CD11b (integrin alpha M, ITGAM) is a leukocyte‐specific receptor marker for granulocytes and was used as an early differentiation marker since it is known to be detectable after 3 days of DMSO differentiation.^[^ ^38^ ^]^ N‐formyl peptide receptor 1 (FPR1) is abundantly expressed on neutrophils and was used as a late differentiation marker that has been shown to appear only after 5 days of differentiation.^[^ ^38^ ^]^ CD11b was analyzed using an APC‐conjugated monoclonal antibody against CD11b and FPR1 was measured using a fluorescent peptide ligand for FPR1 (FLPEP). Both unstained negative controls and isotype controls for the CD11b antibody were used for each time point and each sample and showed no staining (Figure S3, Supporting Information). CD11b (**Figure** **3**A) and FLPEP (Figure 3B) positive cells were quantified by flow cytometry for undifferentiated and day 7 differentiated HL‐60 Bcl‐2 cells illustrating a clear shift in positive cells after 7 days of treatment. Undifferentiated HL‐60 Bcl‐2 cells only expressed a low percentage of CD11b between 4 to 7 days of treatment (15‐20% positive), whereas even after only 4 days of DMSO treatment, the differentiated cells were already at 94% CD11b positive and increased to 98% by day 7 (Figure 3C). Similarly, undifferentiated cells did not express any FLPEP (<1%) while 50% of differentiated HL‐60 Bcl‐2 cells were FLPEP positive by day 7 (Figure 3D). Generally, the cells that were positive for FLPEP overlapped with those that were CD11b positive, which further validated our results (Figure S2B, Supporting Information). Additionally, neutrophil differentiation was further confirmed with gene expression changes of several neutrophil markers by RNASeq analysis (Figure S4A,B, Supporting Information, red stars point out the same surface markers used in flow cytometry). These data validate that HL‐60 Bcl‐2 cells can be effectively differentiated into neutrophil‐like cells within 7 days of 1.3% DMSO treatment.

Since we identified a small percentage of primary neutrophils that produced long and dynamic McTNs (Figure 1), we wanted to determine whether differentiated neutrophils maintained this capability. To do this, HL‐60 Bcl‐2 cells were differentiated for 7 days into neutrophils, tethered onto a TetherChip on each day, and stained with Hoechst to further confirm differentiation via nuclear morphology changes (Figure 3E; Figure S12A, Supporting Information). We observed the expected maturation process, in which the nuclei became more segmented with the earlier stages (day 0–2) having a rounded, single‐lobed nuclei, the middle stage (day 3–4) nuclei started to become indented, and the later stages (day 5–7) being completely multi‐lobed. The same cells were simultaneously stained with a plasma membrane dye, WGA, to assess McTN formation (Figure 3F; Figure S5A, Supporting Information). As the differentiation process progressed, the number of McTN positive cells increased from ≈13% on day 1 to 44% by day 7 (Figure 3G). A small number of HL‐60 cells are known to spontaneously differentiate in culture,^[^ ^39^ ^]^ which could be a reason we see 20% McTN positivity on day 0 (Figure 3G). This aligns with the ≈20% of CD11b positive cells by flow cytometry in the undifferentiated condition (Figure 3C). Additionally, to confirm that these cells could have spontaneously differentiated, the undifferentiated population was stained with CD11b (validated in Figure S5B, Supporting Information) and WGA for visualization (Figure S5C, Supporting Information). The one cell that produces McTNs (green arrow) is also CD11b positive (magenta arrow), whereas the other cells in the field of view are negative for both CD11b and McTNs. This does not mean that every CD11b positive cell will have McTNs, but it does illustrate that the cells producing McTNs are consistently CD11b positive, strengthening the hypothesis that McTNs are formed by differentiated neutrophils. Alternatively, since HL‐60 cells are intrinsically a leukemia cell line, it is entirely possible that the small population of undifferentiated HL‐60 cells with McTNs could have resulted from the tumor origin of the cell line. On the seventh day of DMSO treatment, fully differentiated neutrophils had more numerous and longer McTNs than those at earlier stages of neutrophil development (promyelocyte, myelocyte, metamyelocyte, banded neutrophil) (Figure 3F). To confirm these protrusions were McTNs, immunofluorescence was performed on a TetherChip for day 7 differentiated HL‐60 Bcl‐2 cells and stained for α‐tubulin since McTNs are microtubule‐based protrusions (Figure 3H). The overlap of α‐tubulin and WGA (plasma membrane) staining illustrates that differentiation‐induced neutrophils produce McTNs (white arrows). For the remaining experiments, differentiated HL‐60 Bcl‐2 cells will be referred to as HL‐60 Bcl‐2(N) cells or differentiated neutrophils were used at day 7 of differentiation for functional assays unless indicated otherwise. Overall, we demonstrate that our HL‐60 Bcl‐2(N) model can be used to study neutrophils in vitro and that neutrophil‐like cells produce tubulin‐based McTNs.

### 2.4 Tubulin Post‐Translational Modifications are Enhanced in Differentiated Neutrophils

**Figure 4:**
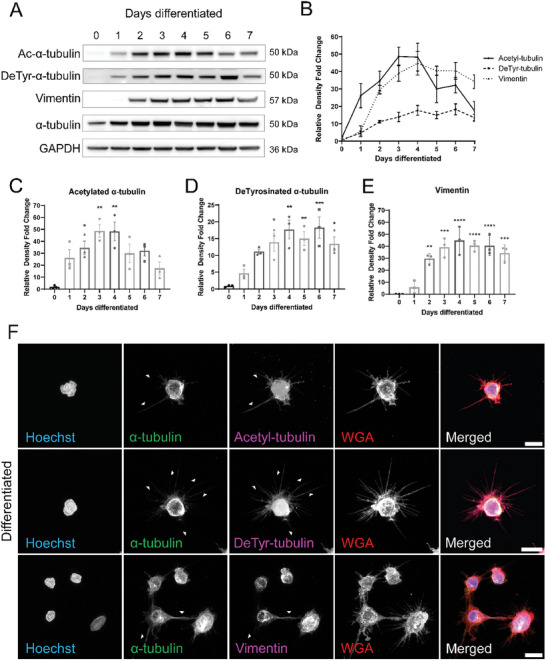
Tubulin post‐translational modifications are enhanced in differentiated neutrophils. A) Western blot analysis of HL‐60 Bcl‐2(N) cells differentiated with 1.3% DMSO over 7 days probed for acetylated α‐tubulin, detyrosinated α‐tubulin, α‐tubulin, vimentin, and GAPDH. B) Densitometry measurements normalized to α‐tubulin levels for acetyl and detyrosinated α‐tubulin and GAPDH levels for vimentin. C) Densitometry statistical analysis for acetylated α‐tubulin protein expression. Error bars indicate mean ± standard error of mean, n = 3. *p < 0.05; **p < 0.01 versus day 0 (One‐way ANOVA with Bonferroni post‐test). D) Densitometry statistical analysis for detyrosinated α‐tubulin protein expression. Error bars indicate mean ± standard error of mean, n = 3. *p < 0.05; **p < 0.01; ***p < 0.001 versus day 0 (One‐way ANOVA with Bonferroni post‐test). E) Densitometry statistical analysis for vimentin protein expression. Error bars indicate mean ± standard error of mean, n = 3. **p < 0.01; ***p < 0.001; ****p < 0.0001 versus day 0 (One‐way ANOVA with Bonferroni post‐test). F) Immunofluorescent images of tethered and fixed day 7 differentiated neutrophils stained with Hoechst, α‐tubulin, WGA and either acetylated α‐tubulin, detyrosinated α‐tubulin or vimentin. Images were taken at 60x magnification using a Nikon Ti2‐E inverted microscope with a Nikon AX‐R confocal system. Images were denoised in a post‐processing step using NIS Elements. Scale bar = 10 µm.

In order to determine the mechanism of McTN formation, HL‐60 Bcl‐2 cells were differentiated into neutrophils over 7 days and assessed for tubulin post‐translational modifications and intermediate filaments that were previously found to be enhanced in McTNs (**Figure** **4**A,B). Acetylated α‐tubulin modification on lysine 40 (K40) increased to a maximum level of ≈50‐fold by day 3 of differentiation (Figure 4C), while detyrosinated α‐tubulin increased to its maximum level of ≈18‐fold by day 4 then plateaued, remaining high (Figure 4D). Vimentin was also upregulated by ≈45‐fold by day 5 and remained high until day 7 (Figure 4E). These results demonstrate the upregulation of α‐tubulin post‐translational modifications begin to increase by day 1 and precede the increase in vimentin, an intermediate filament, which starts to increase only by day 2, suggesting a potential stepwise regulation of initial post‐translational tubulin modifications prior to an upregulation in vimentin expression for McTN formation, which was previously unknown. Immunofluorescent imaging of day 7 differentiated (Figure 4F; Figure S6B, Supporting Information) neutrophils illustrates that the McTNs on the differentiated neutrophils are primarily composed of detyrosinated α‐tubulin and acetylated α‐tubulin with some vimentin staining as well. Compared to the differentiated neutrophils, the undifferentiated HL‐60 cells did not have any McTNs and therefore had less acetylated and detyrosinated α‐tubulin and vimentin staining as well (Figure S6A, Supporting Information). These data further confirm that the membrane protrusions we detect after differentiation into neutrophils are composed of the same α‐tubulin post‐translational modifications and intermediate filament, vimentin, previously seen on McTNs from epithelial tumor cells.^[^ ^15^, ^17^, ^19^ ^]^ For the remaining experiments, differentiated neutrophils were used at day 7 of DMSO differentiation for functional assays.

### 2.5 Differentiation into Neutrophils Induces Cluster Formation, Migration, and Reattachment

**Figure 5:**
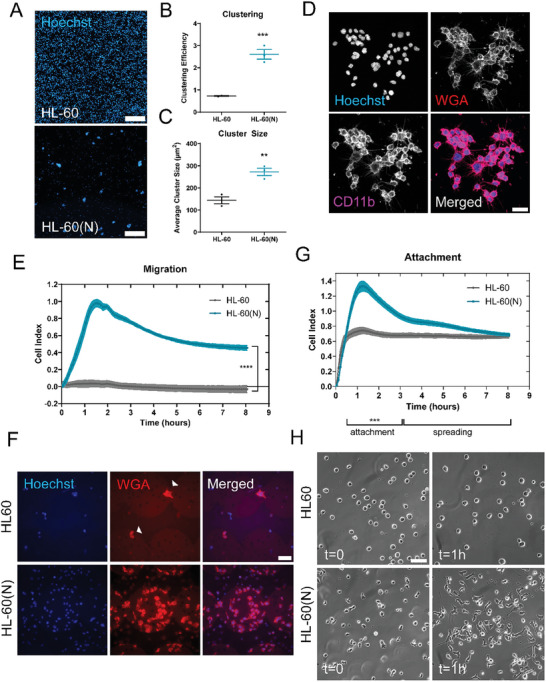
Differentiation into neutrophils induces cluster formation, migration, and attachment. A) Representative images of undifferentiated (top panel) and day 7 differentiated (bottom panel) HL‐60 Bcl‐2 cells that were allowed to cluster for 24 h and then tethered onto TetherChips and stained with Hoechst. Scale bar = 500 µm. B) Clustering efficiency analysis on the number of clusters that formed over 24h. Individual values at t = 0 were divided by respective t = 24 h number of clusters for each condition. Error bars indicate ± standard error of mean, n = 3. ***p < 0.001 versus undifferentiated HL‐60 Bcl‐2 (Two‐tailed t‐test). C) Average cluster size measurements of clusters formed after 24 h. Error bars indicate mean ± standard error of mean, n = 3. **p < 0.01 versus HL‐60 Bcl‐2 (Two‐tailed t‐test). D) Representative image of day 7 differentiated neutrophil cluster stained with Hoechst, WGA, and CD11b. Images were taken at 60x magnification using a Nikon Ti2‐E inverted microscope with a Nikon AX‐R confocal system. Images were denoised in a post‐processing step using NIS Elements. Scale bar = 20 µm. E) Representative graph of migration efficiency of HL‐60 Bcl‐2 cells versus the day 7 differentiated neutrophils (HL‐60(N)) toward fMLP over the course of 8 h on an xCelligence RTCA system. Error bars indicate ± standard deviation, n = 3. ****p < 0.0001 versus HL‐60 Bcl‐2 (from 0.3 to 8 h, two‐way ANOVA with Bonferroni post‐test). F) Representative images of the membrane of an xCelligence transwell cartridge stained with Hoechst and WGA illustrating HL‐60 or HL‐60(N) cells after migration through the pores. Images were taken with a Nikon Ti2‐E inverted microscope at 60x magnification. Scale bar = 50 µm. White arrows represent cells that have migrated through the pores of the membrane and attached to the other side. The small black dots, which can most easily be seen on the WGA channel are the membrane pores that the cells must squeeze through to migrate to the other side of the cartridge. G) Representative graph of reattachment efficiency of HL‐60 cells versus HL‐60(N) cells. Error bars indicate ± standard deviation, n = 3. ****p < 0.0001 versus HL‐60 Bcl‐2 (from 0.5 to 6 h, two‐way ANOVA with Bonferroni post‐test). H) Representative phase contrast still frames of HL‐60 (top panels) or HL‐60(N) (bottom panels) cells attaching onto fibronectin‐coated 24‐well plates at t = 0 (left panels) and t = 1 h (right panels). Images were taken at 60x magnification on a Nikon Ti2‐E inverted microscope with a stage‐top Tokai‐hit incubator chamber. Scale bar = 50 µm.

Our lab has previously shown that tumor cell McTNs promote phenotypes required in multiple steps of the metastatic cascade such as aiding in homotypic clustering, reattachment, and migration of tumor cells.^[^ ^16^, ^23^ ^]^ To determine whether these neutrophil protrusions function as McTNs, undifferentiated HL‐60 cells, and differentiated neutrophils were allowed to cluster at the same density for 24 h and then tethered onto TetherChip for visualization and analysis (**Figure** **5**A,D; Figure S7A,B, Supporting Information). Our data revealed that differentiation into neutrophils significantly enhanced both clustering efficiency (Figure 5B) and average cluster size (Figure 5C) compared to the non‐differentiated HL‐60 control. Similarly, the differentiated neutrophils had a substantial upregulation in migration over 8 h whereas the undifferentiated HL‐60s lacked any ability to migrate toward the same chemoattractant, N‐formylmethionine‐leucyl‐phenylalanine (fMLP) (Figure 5E; Figure S7C, Supporting Information). Representative images of the underside of the microporous membrane on the migration cartridge further demonstrated only the differentiated neutrophils were able to migrate through the pores (Figure 5F; Figure S7D, Supporting Information). White arrows represent cells that have migrated through the pores of the membrane and attached to the other side. The small black dots, which can most easily be seen on the WGA channel are the membrane pores that the cells must squeeze through to migrate to the other side of the cartridge. Similarly, differentiation into neutrophils was also accompanied by a significant increase in initial reattachment onto a fibronectin‐coated cartridge whereas spreading was not as affected (Figure 5G; Figure S7E, Supporting Information). To visualize and confirm reattachment, time‐lapse microscopy was performed on undifferentiated and differentiated HL‐60 Bcl‐2 cells over the course of 24 h (Figure 5H; Videos S1 and S2, Supporting Information, the 1‐h time point was shown as a still image because that was the peak of reattachment). The undifferentiated cells remained suspended even over fibronectin‐coated tissue‐culture plates throughout the experiment (Figure 5H top two panels) while the differentiated neutrophils had almost fully reattached to the fibronectin by 1 h (Figure 5H lower two panels). These results reveal that differentiation into neutrophils enhances McTN formation, which promotes several McTN‐mediated phenotypic behaviors that strongly correlate with metastatic properties such as homotypic clustering, migration, and reattachment.

### 2.6 Vinorelbine Treatment of Differentiated Neutrophils Disrupts Microtentacle Formation and Microtentacle‐Supported Phenotypes

**Figure 6:**
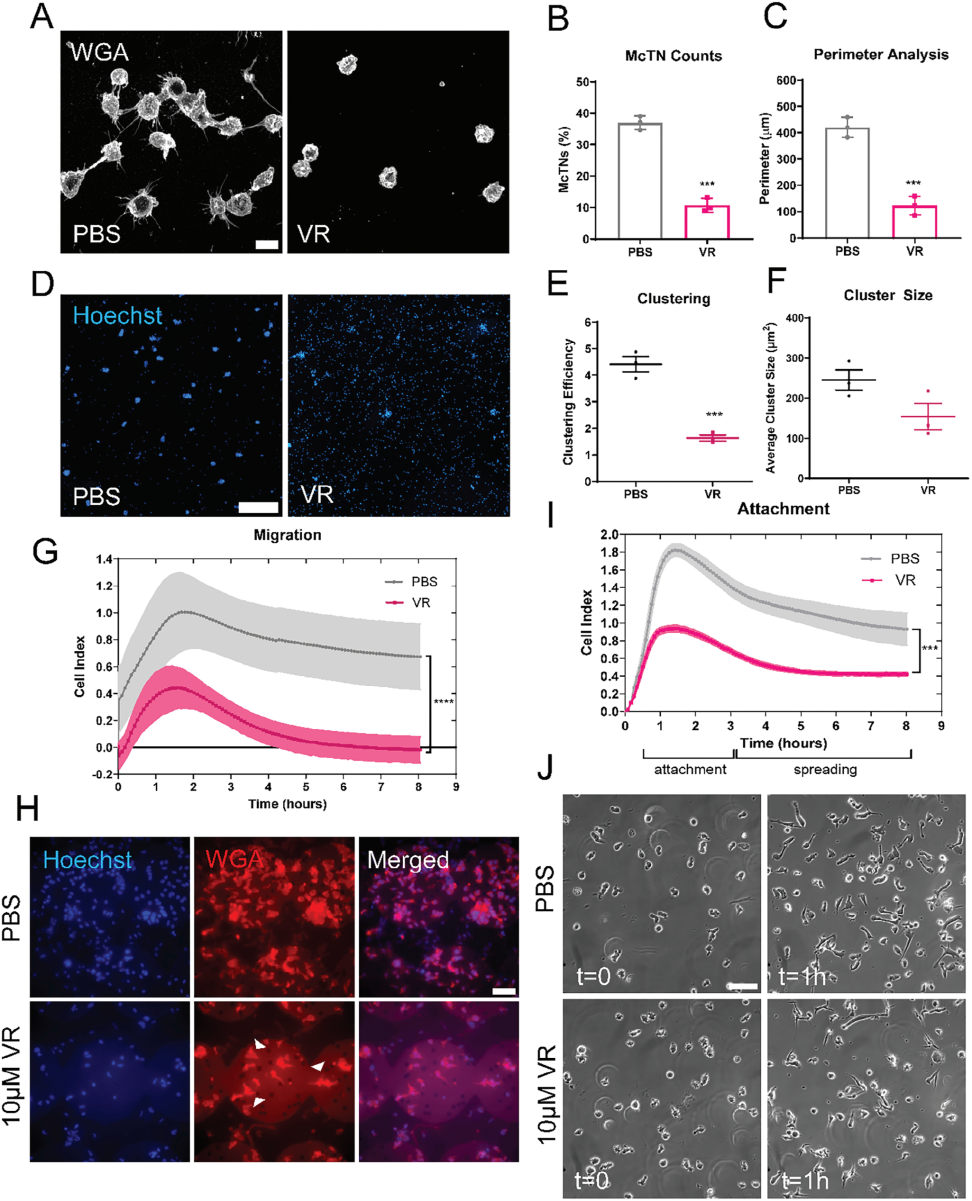
Vinorelbine treatment of differentiated neutrophils disrupts McTN formation and McTN‐supported phenotypes. A) Immunofluorescence images of tethered and fixed day 7 differentiated neutrophils treated with Vinorelbine (10 µm, VR) for 1 h, stained with WGA. Images were taken at 60x magnification using a Nikon Ti2‐E inverted microscope with a Nikon AX‐R confocal system. Scale bar = 10 µm. B) McTN quantification of day 7 differentiated neutrophils treated with PBS (vehicle) versus 10 µm VR tethered on TetherChip. Data represents quantification of McTN frequency from three independent experiments with 100 cells counted for each. Data are shown as mean ± standard deviation, n = 3. ***p < 0.001 versus PBS (Two‐tailed t‐test). Scale bar = 500 µm. C) Quantification of the perimeter of differentiated neutrophils treated with either PBS or 10 µm VR analyzed by ImageJ. Data are shown as mean ± standard deviation, n = 3. ***p < 0.001 versus PBS (Two‐tailed t‐test). D) Representative images of differentiated neutrophils cells treated with either PBS (left panel) or 10 µm of VR (right panel) that were allowed to cluster for 24 h and then tethered onto TetherChips. Scale bar = 500 µm. E) Clustering efficiency analysis on the number of clusters that formed over 24 h. Individual values at t = 0 were divided by respective t = 24 h number of clusters for each condition. Error bars indicate mean ± standard error of mean, n = 3. ***p < 0.001 versus PBS treated (Two‐tailed t‐test). F) Average cluster size measurements of clusters formed after 24 h. Error bars indicate mean ± standard error of mean, n = 3. G) Representative graph of migration efficiency of day 7 differentiated neutrophils treated with either PBS or 10 µm VR toward fMLP over the course of 8 h. Error bars indicate mean ± standard deviation, n = 3. ****p < 0.0001 versus PBS‐treated (from 0.6 to 8 h, two‐way ANOVA with Bonferroni post‐test). H) Representative images of the membrane of an xCelligence transwell cartridge stained with Hoechst and WGA illustrating PBS‐ or 10 µm Vinorelbine‐treated differentiated neutrophils after migration through the pores. Images were taken with a Nikon Ti2‐E inverted microscope at 60x magnification. Scale bar = 50 µm. White arrows represent cells that have migrated through the pores of the membrane and attached to the other side. The small black dots, within the larger horizontally aligned circles, which can most easily be seen on the WGA channel are the membrane pores that the cells must squeeze through to migrate to the other side of the cartridge. I) Representative graph of reattachment efficiency of day 7 differentiated neutrophils treated with PBS or 10 µm VR. Error bars indicate mean ± standard deviation, n = 3. ****p < 0.0001 versus PBS‐treated (from 1.6 to 8 h, two‐way ANOVA with Bonferroni post‐test). J) Representative phase contrast still frames of differentiated neutrophils treated with PBS (top panels) or 10 µm VR (bottom panels) attaching onto fibronectin‐coated plates at t = 0 (left panels) and t = 1 h (right panels). Images were taken at 60x magnification on a Nikon Ti2‐E inverted microscope with a stage‐top Tokai‐hit incubator chamber. Scale bar = 50 µm.

Since it was confirmed that the protrusions formed after differentiation into neutrophils were α‐tubulin‐derived McTNs, we examined whether these McTNs could be inhibited using the tubulin depolymerizing agent, Vinorelbine. Both undifferentiated HL‐60 Bcl‐2 cells and differentiated neutrophils were treated for 1 h with 10 µm Vinorelbine and acetylated and detyrosinated α‐tubulin protein expression was measured (Figure S8A, Supporting Information). Both α‐tubulin post‐translational modifications increased after differentiation, however there was no effect after drug treatment, which has been previously reported.^[^ ^23^ ^]^ This is unsurprising as Vinorelbine prevents tubulin polymerization by binding to microtubular proteins in the mitotic spindle during metaphase to inhibit cell division; so it is not expected to modulate the amount of tubulin protein expression, but rather change its structure.^[^ ^23^ ^]^ Cell viability was also measured to verify that the drug treatment was not affecting the integrity of the cells (Figure S8B, Supporting Information). Vinorelbine treatments of up to 25 µm did not have a significant effect on viability of the differentiated neutrophils compared to the phosphate buffered saline (PBS)‐treated negative control. Staurosporine‐treated differentiated neutrophils were used as a positive control for decreased cell viability. Moving forward with the previously established concentration of 10 µm,^[^ ^23^ ^]^ differentiated neutrophils were treated with Vinorelbine and then tethered onto TetherChip for visualization and analysis (**Figure** **6**A). McTN positivity (Figure 6B) and cell perimeter, an orthogonal approach to quantify McTNs (Figure 6C), were both significantly decreased after Vinorelbine treatment compared to the PBS‐treated control. These data demonstrate that treatment with an FDA‐approved tubulin depolymerizing agent efficiently reduces McTN formation on differentiated neutrophils without affecting cellular viability. We have previously shown that McTNs on tumor cells are insensitive to actin inhibition via Cytochalasin D treatment,^[^ ^16^ ^]^ stabilized with Paclitaxel treatment^[^ ^43^ ^]^ and reduced via Tetracaine treatment.^[^ ^44^ ^]^ To further validate these protrusions are McTNs, differentiated neutrophils were treated with Tetracaine, Paclitaxel and Cytochalasin D, tethered and stained with WGA to look at McTN phenotypes (Figure S9A, Supporting Information). As expected, Tetracaine inhibited McTNs and drastically reduced cell perimeter, Paclitaxel increased McTNs and Cytochalasin D had no effect on either McTN frequency or perimeter analysis (Figure S9B and S9C). The combination of the tubulin post‐translational modifications and vimentin expression, phenotypic characteristics, and pharmacological modulation of membrane protrusions verifies these protrusions produced on the differentiated neutrophils are McTNs.^[^ ^15^, ^17^, ^19^, ^45^ ^]^

Because Vinorelbine treatment decreased McTNs, we next wanted to determine whether inhibiting McTN formation on differentiated neutrophils could also impede the upregulation of homotypic clustering, migration, and reattachment that was caused by neutrophil differentiation. This would validate that McTNs are necessary to promote these metastatic behaviors and confirm the results were not just an artifact of differentiation. Differentiated neutrophils were allowed to cluster for 24 h in either PBS‐ or 10 µm Vinorelbine‐containing media and tethered onto TetherChip for visualization and analysis (Figure 6D; Figure S8C, Supporting Information). Clustering efficiency (Figure 6E) was significantly decreased, and average cluster size (Figure 6F) was also downregulated with Vinorelbine treatment. Migration (Figure 6G,H; Figure S8D,E, Supporting Information) and reattachment (Figure 6I,J; Figure S8F, Supporting Information) were also significantly reduced after Vinorelbine treatment compared to the PBS‐treated control. Live reattachment time‐lapse images illustrated almost complete reattachment of the PBS‐treated differentiated neutrophils to fibronectin by 1 h (Figure 6J, top panels) compared to the Vinorelbine‐treated cells that only partially reattached by 1 h (Figure 6J, bottom panels; Videos S3 and S4, Supporting Information). These results confirm that McTNs play a substantial role in homotypic clustering, migration, and reattachment to ECM. Thus, by inhibiting McTNs with a tubulin‐destabilizing chemotherapeutic agent, we can reduce these phenotypic behaviors that correlate with metastatic properties. Importantly, these data are clinically relevant since Vinorelbine is an already FDA‐approved drug, therefore, using it in combination with other therapies may help patients with existing primary tumors avoid progression to metastasis by reducing McTNs on any CTCs.

### 2.7 Differentiated Neutrophils and Tumor Cells form Heterotypic Clusters that can be Disrupted with Vinorelbine

**Figure 7:**
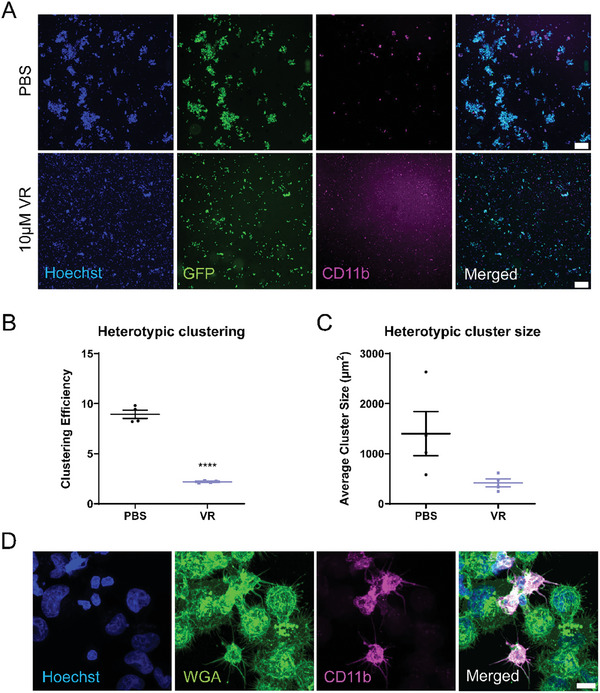
Differentiated neutrophils and tumor cells form heterotypic clusters that can be inhibited with Vinorelbine. A) Representative images of PBS‐(top panel) versus Vinorelbine (10 µm, bottom panel)‐treated tumor cell‐day 7 differentiated neutrophil heterotypic clusters that were allowed to cluster for 6 h under low‐attach conditions and then tethered onto TetherChips and stained with Hoechst. Scale bar = 300 µm. B) Clustering efficiency analysis on the number of heterotypic clusters that formed over 6 h. Individual values at t = 0 were divided by respective t = 6 h number of clusters for each condition. Error bars indicate mean ± standard error of mean, n = 4. ****p < 0.0001 versus PBS treated (Two‐tailed t‐test). C) Average cluster size measurements of clusters formed after 6 h. Error bars indicate mean ± standard error of mean, n = 4. D) Representative image of heterotypic clusters stained with Hoechst, WGA, and CD11b. Images were taken at 60x magnification using a Nikon Ti2‐E inverted microscope with a Nikon AX‐R confocal system. Images were denoised in a post‐processing step using NIS Elements. Scale bar = 10 µm.

It has been previously shown that neutrophils and breast tumor cells interact and cluster together in the bloodstream to enhance metastasis in mouse models.^[^ ^7^ ^]^ To further prove that McTNs may play a role in or mediate this heterotypic clustering, GFP‐expressing MDA‐MB‐231TD (tumor‐derived)^[^ ^23^ ^]^ cells and differentiated neutrophils stained with CD11b were co‐cultured in either PBS‐ or Vinorelbine‐containing media and allowed to cluster for 6 h under low‐attach conditions (**Figure** **7**A; Figure S10A, Supporting Information). Heterotypic clusters formed efficiently between the tumor cells and differentiated neutrophils and Vinorelbine treatment inhibited heterotypic clustering efficiency (Figure 7B) as well as reduced average cluster size (Figure 7C). Representative image of a heterotypic cluster illustrated that McTNs on the differentiated neutrophils (CD11b+ cells, magenta) can wrap around the adjacent tumor cells to form clusters (Figure 7D). These results confirm that tumor cells and differentiated neutrophils form heterotypic clusters that can be inhibited pharmacologically via targeting McTNs with Vinorelbine.

### 2.8 Differentiated Neutrophil‐Tumor Cell Heterotypic Clustering Enhances Migration

**Figure 8:**
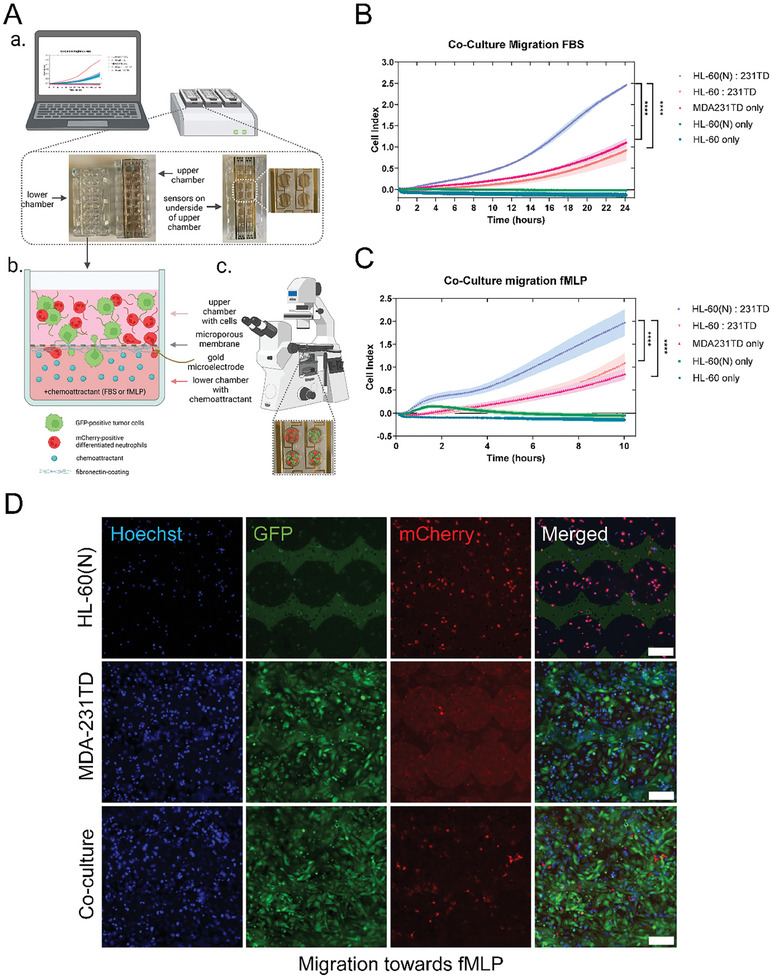
Co‐culturing day 7 differentiated neutrophils with tumor cells enhance migration. A) Diagram of xCelligence co‐culture migration assay. a) Schematic of the migration cartridge with an upper and lower chamber and a microporous membrane in between, which is coated with gold microelectrodes on the underside to sense the cells migrating through. b) Schematic of an individual well in the cartridge where the cells are seeded after co‐culturing and allowed to migrate through the pores over time. c) The top and bottom chamber can be detached following the termination of the experiment and the top chamber can be fixed and mounted with a coverslip to image on the microscope. B) Representative graph of migration efficiency of undifferentiated HL‐60 Bcl‐2 cells only, differentiated neutrophils only, MDA‐MB‐231TD cells only, a 1:1 co‐culture of undifferentiated HL‐60 Bcl‐2 cells and MDA‐MB‐231TD cells and a 1:1 co‐culture of differentiated neutrophils and MDA‐MB‐231TD cells toward 10% FBS (tumor cell attractant) over the course of 24 h. Error bars indicate mean ± SD, each experiment was performed in triplicates. ****, *p* < 0.0001 of tumors cells only versus differentiated neutrophil: tumor cell co‐culture from 8.5 to 24 h and undifferentiated HL‐60 Bcl‐2 cells: tumor cell co‐culture versus differentiated neutrophil: tumor cell co‐culture from 6 to 24 h (Two‐way ANOVA with Bonferroni post‐test). C) Representative graph of migration efficiency of undifferentiated HL‐60 Bcl‐2 cells only, differentiated neutrophils only, MDA‐MB‐231TD cells only, a 5:1 co‐culture of undifferentiated HL‐60 Bcl‐2 cells and MDA‐MB‐231TD cells and a 5:1 co‐culture of differentiated neutrophils and MDA‐MB‐231TD cells toward 200 nm fMLP (neutrophil attractant) over the course of 8 h. Error bars indicate mean ± SD, each experiment was performed in triplicate. ****, *p* < 0.0001 of tumors cells only versus differentiated neutrophil: tumor cell co‐culture from 4 to 8 h and differentiated neutrophil: tumor cell coculture versus undifferentiated HL‐60 Bcl‐2 cell: tumor cell co‐culture from 1.5 to 8 h (Two‐way ANOVA with Bonferroni post‐test). D) Fluorescent images of a fibronectin‐coated, formaldehyde‐fixed, Hoechst‐stained xCelligence migration CIM cartridge with GFP‐labeled MDA‐MB‐231TD tumor cells and mCherry‐labeled differentiated neutrophils that migrated through pores toward 200 nm fMLP. Images were taken at 20x magnification using a Nikon Ti2‐E inverted microscope. Scale bar = 100 µm.

To determine whether the co‐culture clusters confer any migration advantages over single cells alone, undifferentiated HL‐60 Bcl‐2 cells alone, differentiated neutrophils alone, MDA‐MB‐231TD (tumor‐derived)^[^ ^23^ ^]^ cells alone, and either a co‐culture of MDA‐MB‐231TD cells and mCherry‐labeled undifferentiated HL‐60 Bcl‐2 cells or a co‐culture of MDA‐MB‐231TD cells and mCherry‐labeled differentiated neutrophils were allowed to cluster overnight and plated for migration toward 10% FBS, a cancer cell chemoattractant, or fMLP, a neutrophil chemoattractant (**Figure** **8**A). As expected, the unmixed, pure population of undifferentiated HL‐60 Bcl‐2 cells and differentiated neutrophils did not migrate at all toward FBS (Figure 8B; Figures S11A,B, Supporting Information, green and teal lines) since neutrophils can only sense chemoattractants such as leukotrienes (LTB_4_), chemokines (IL8), anaphylatoxins (C3a and C5a) as well as formyl peptides (fMLP).^[^ ^46^ ^]^ The unmixed, pure population of MDA‐MB‐231TD cells migrated efficiently within 24 h (pink line), which was also expected since FBS is a known chemoattractant for these tumor cells. However, MDA‐MB‐231TD cell migration was dramatically enhanced by the addition of the differentiated neutrophils with the migration of the cell combination doubling that of the tumor cells alone (Figure 8B; Figure S11A,B, Supporting Information, lavender line). Given that both the undifferentiated HL‐60 Bcl‐2 cells and the differentiated neutrophils did not migrate at all by themselves toward FBS, this suggests that the tumor cells could potentially be transporting the differentiated neutrophils as heterotypic clusters through the pores to account for the increase in cell index. Importantly, the co‐culture between undifferentiated HL‐60 Bcl‐2 cells and tumor cells (Figure 8B; Figure S11A,B, Supporting Information, coral line) did not affect migration as the migration was similar to the tumor cells alone. These results indicate that the enhanced migration is specifically due to an effect of the differentiated neutrophils. To verify that heterotypic cell clusters migrate through the membrane, complementary co‐culture experiments using fMLP as the chemoattractant were additionally performed. The unmixed, pure population of differentiated neutrophils efficiently migrated toward fMLP, which was seen in earlier figures (Figures 5 and 6), with a peak migration capacity of around 1—2 h (Figure 8C; Figure S11C,D, Supporting Information, green line). The co‐culture of tumor cells and differentiated neutrophils again resulted in a significant increase in migration toward fMLP over the span of 8 h as compared to the tumor cells alone. Additionally, similar to migration toward FBS, the co‐culture between the undifferentiated HL‐60 Bcl‐2 cells and tumor cells did not result in an increase in migration toward fMLP further supporting the effects of specifically the differentiated neutrophils. Representative fluorescent images of migration toward fMLP (Figure 8D) and FBS (Figure S11E, Supporting Information) illustrate the underside of the migration cartridge after the cells migrated through the pores (black dots). Importantly, these results suggest that heterotypic clusters have an advantage over both single cells and homotypic clusters because they can respond to multiple stimuli (Ex: FBS and fMLP) rather than to just one or the other. This would undoubtedly benefit a tumor cell traveling through the vasculature trying to make its way to a secondary site. Overall, these results confirm that differentiated neutrophils and tumor cells interact, possibly mediated by McTNs, to enhance migration.

## 3 Discussion

Emerging evidence indicates that heterotypic neutrophil‐CTC clusters found in the bloodstream of patients with metastatic breast cancer correlate to reduced progression‐free survival compared to both single CTCs and homotypic CTC clusters.^[^ ^7^ ^]^ Our lab has also shown that functionally, McTNs aid in tumor cell reattachment to the endothelial cells within the vessel wall and homotypic and heterotypic aggregation.^[^ ^15^, ^23^ ^]^ Our previous work of identifying and characterizing McTNs was performed on various epithelial tumor cells, however, given that heterotypic clusters of tumor and immune cells have been identified in the bloodstream of cancer patients, we aimed to determine whether immune cells, specifically neutrophils, are additionally able to produce McTNs. Utilizing our microfluidic TetherChip technology, we explored McTN formation on neutrophils to determine if they had similar functional phenotypes. For the first time, this study demonstrated that both primary and differentiated neutrophils form unique McTNs composed of detyrosinated and acetylated α‐tubulin and vimentin that facilitated homotypic clustering, migration, and reattachment. Using the FDA‐approved microtubule depolymerizing agent, Vinorelbine, the McTNs produced by differentiated neutrophils were inhibited, thereby reducing homotypic clustering, migration and the reattachment potential of differentiated neutrophils. Finally, we revealed that co‐culturing differentiated neutrophils and tumor cells resulted in heterotypic clustering that enhanced migration capacity and that these heterotypic clusters can also be disrupted with Vinorelbine treatment.

Several immune cell types, including neutrophils, macrophages, T cells, B cells, and natural killer cells are present in the tumor microenvironment.^[^ ^47^ ^]^ Beyond these interactions at the primary tumor site, it has now been well‐established that immune cells are also active during tumor progression, such as in the circulation and in secondary metastatic sites.^[^ ^48^ ^]^ Until recently, the role and functions of circulating neutrophils have been significantly overlooked in metastasis even though neutrophils comprise a substantial proportion of the tumor‐infiltrating lymphocytes as well as the majority of white blood cells in circulation. It is inevitable that a circulating tumor cell will interact with a neutrophil while traveling through the bloodstream. Our lab previously published that when tumor cells encounter non‐adherent environments like the bloodstream, they produce McTNs that are primarily composed of α‐tubulin and aid in clustering, migration, and reattachment.^[^ ^16^, ^23^ ^]^ McTNs positively correlate with tumor aggressiveness, are present on tumor cells isolated from patient samples,^[^ ^49^ ^]^ and enhance metastasis in four different genetic models.^[^ ^24^, ^25^, ^26^, ^27^, ^28^ ^]^ Tau, a microtubule‐associated protein, directly induced McTNs, and its overexpression enhanced tumor cell retention in the lungs of mice.^[^ ^24^ ^]^ Dominant negative c‐Src, a tyrosine‐protein kinase, also upregulated McTNs and enhanced trapping and retention of CTCs in lung capillaries of mice.^[^ ^25^ ^]^ Loss of obscurin, a sarcomeric signaling protein, increased McTN formation via decreased RhoA^[^ ^27^ ^]^ and drastically enhanced breast tumor formation and metastasis in mouse models.^[^ ^28^ ^]^ Finally, loss of PTEN increased tumor cell survival, McTNs, and reattachment via cofilin activation.^[^ ^26^ ^]^ Whether or not other cell types besides epithelial tumor cells formed McTNs, however, remained unknown. Taking advantage of our TetherChip technology and its ability to spatially immobilize cells on an optically clear nanosurface while maintaining non‐adherent phenotypes, we illustrate here that both primary (Figure 1) and differentiated neutrophils produce McTNs that positively correlate with differentiation status (Figure 3). As differentiation progressed from a promyelocytic stage to a mature neutrophil, the quantity and length of McTNs gradually increased. As expected, the McTNs themselves were mainly composed of detyrosinated and acetylated α‐tubulin, however, increases in vimentin were also evident in differentiated neutrophils (Figure 4). Our study demonstrates for the first time, that McTNs exist not only in epithelial tumor cells but also in neutrophils as well. This has significant implications for elucidating a possible mechanism of tumor cell‐immune cell interaction in the vasculature and a potential strategy for targeting these interactions to reduce metastasis in patients.

The importance of CTC clusters has become increasingly evident over the past decade. Although they only encompass a small fraction (3‐6%) of total CTC events found in the blood vasculature of patients or mouse models,^[^ ^50^ ^]^ the presence of CTC clusters has been correlated to decreased survival and elevated metastasis. It was shown that about 40–50% of patients with metastatic breast cancer have at least one CTC cluster.^[^ ^50^ ^]^ Because of this, it has been suggested that the formation of CTC clusters could be one of the key drivers to initiate the metastatic process. More recently, studies have shown that CTC clusters can be heterogeneous, meaning that tumor cells can form clusters with other cell types such as white blood cells and stromal cells.^[^ ^51^ ^]^ Emerging research now suggests that heterotypic clusters found in the bloodstream have the potential to be even more metastatic than homotypic clusters.^[^ ^52^ ^]^ Previously, we have shown that McTNs aid in homotypic clustering in cancer cells, but never extrapolated our findings to any other cell type.^[^ ^23^ ^]^ In this study, utilizing TetherChip, we reveal that McTNs on differentiated neutrophils not only facilitate homotypic clustering (Figure 5), but also aid in heterotypic clustering to promote migration with tumor cells (Figures 7 and 8). However, the molecular mechanisms by which McTNs promote clustering are still largely unknown. Whether McTN‐mediated clustering depends on certain molecules like adhesion or desmosomal proteins or whether they indiscriminately bind to lipids on plasma membranes on nearby cells are questions still under investigation. The simplest model is that McTNs simply extend the cell surface, and the existing cell surface receptors can then enable a tighter clustering. Although a receptor specific to McTNs has not been found yet, an efficient microtubule‐based transport system exists within McTNs to potentially transport receptors to the McTN ends.

Targeting CTC clusters has the potential to be highly impactful in patients with advanced‐stage metastatic disease. Currently, there is only one clinical trial specifically targeting CTC clusters using a cardiac glycoside, Digoxin, to attempt to disrupt clusters in breast cancer patients (NCT03928210). Digoxin, an FDA‐approved therapy to treat heart failure, works by inhibiting the Na+/K+ ATPase, causing an increase in intracellular sodium levels which in turn increases intracellular calcium concentrations by decreasing the activity of the sodium‐calcium exchanger. In the study, the authors showed that Digitoxin, a cardiac glycoside that is similar in structure to digoxin, reduced cluster size, disrupted clusters into single cells and demethylated stemness genes, ultimately reducing metastasis.^[^ ^7^ ^]^ Interestingly, in a drug screen from the same study assessing compounds that could dissociate CTC clusters, the authors found that in addition to the Na+/K+ ATPase inhibitors, tubulin inhibitors also consistently led to a significant decrease in average cluster size even at the lowest concentration tested. However, they did not further investigate these tubulin drugs as possible therapeutic approaches leaving a potential gap warranting further investigation, especially since many tubulin inhibitor drugs are already FDA‐approved for cancer treatment. This clinical trial has recently concluded as of December 2023 and the results are being eagerly awaited. Additionally, many studies have demonstrated that VCAM‐1 and ICAM‐1/Mac‐1 are important for these heterotypic interactions, however, there are currently no FDA‐approved specific therapies for these adhesion molecules, whereas there are already many FDA‐approved microtubule disruptors. Our lab has previously shown that Vinorelbine, an FDA‐approved tubulin destabilizing drug, reduced McTNs, tumor cell clustering, and metastasis in mouse models.^[^ ^23^ ^]^ Vinorelbine, currently approved to treat breast cancer and non‐small cell lung cancer, is a vinca alkaloid agent that inhibits microtubule polymerization. Here, we demonstrate that similarly to breast cancer cells, Vinorelbine inhibits McTNs on differentiated neutrophils (Figure 6) resulting in a reduction of metastatic phenotypes such as homotypic clustering, migration, and reattachment in differentiated neutrophils (Figure 6). It is hypothesized that neutrophils help CTCs survive and migrate through the vasculature to seed at secondary sites by clustering with them. We illustrate that heterotypic clusters form between tumor cells and differentiated neutrophils (Figure 7) and these clusters can be disrupted with Vinorelbine. We also demonstrated that co‐culturing tumor cells and differentiated neutrophils resulted in enhanced migration, most likely attributed to a physical interaction between the cells, presumably mediated by McTNs, since differentiated neutrophils are completely unable to migrate toward FBS in the absence of the tumor cells (Figure 8). Altogether, these results demonstrate that the McTNs on neutrophils may play a role in mediating heterotypic clustering with tumor cells. Disrupting McTNs with microtubule inhibitors could potentially be given as a neoadjuvant therapy to prevent either homotypic or heterotypic CTC clusters from forming thereby reducing the metastatic potential of breast cancer cells.

## 4 Conclusion

Although there are many active clinical trials examining the prognostic effects of homotypic and heterotypic CTC clusters, there are far fewer investigating potential therapies to directly target and reduce the presence of CTC clusters. Disrupting these clusters therapeutically seems reasonable; however, it will be best to ensure that by breaking CTCs apart from each other or from other cells we do not enhance their metastatic capabilities in the process. Multiple standard‐of‐care treatments for the primary tumor can induce CTC shedding,^[^ ^53^ ^]^ therefore anti‐clustering therapies will likely be most effective during the treatment of the primary tumor to help ensure that any shed CTCs do not metastasize efficiently. CTC clusters have been a topic of interest over the past few years due to their significantly enhanced metastatic capacity. We show for the first time that TetherChip can be applied across many different cell types and that, in addition to tumor cells, primary and differentiated neutrophils also form McTNs. McTNs on differentiated neutrophils also mediated phenotypes associated with metastatic behaviors such as clustering, reattachment, and migration, and by inhibiting McTNs with Vinorelbine, these phenotypes were abolished. Lastly, we demonstrate that heterotypic clusters efficiently formed between differentiated neutrophils and tumor cells to enhance migration and that these heterotypic clusters could also be reduced with Vinorelbine. Taken together, this work highlights that targeting neutrophil McTNs with FDA‐approved anti‐microtubule therapies could inhibit cluster formation with tumor cells and help reduce neutrophil‐CTC cluster metastatic efficiency to improve patient prognosis and yield more successful clinical outcomes.

## 5 Experimental Section

### Reagents and Antibodies

DMSO Hybri‐Max (Cat: D2650), Tetracaine hydrochloride (Cat: T7508‐5g) and Cytochalasin D (Cat: C8273‐1mg) were purchased from Sigma, Vinorelbine Tartrate (Cat: 1957‐5) from BioVision, and Paclitaxel (Cat: BML‐T104‐0005) from Enzo. Bcl‐2 antibody (Cat: 15071S) was purchased from Cell Signaling Technologies, DeTyrosinated α‐tubulin (Cat: ab48389) from Abcam or from RevMAb Biosciences (Clone RM444, Cat: 31‐1335‐00), Acetylated α‐tubulin (lys40) (Cat: 5335S) from Cell Signaling Technologies, α‐tubulin (Cat: T6199) from Sigma, Vimentin (EPR3776) (Cat: ab92547) from Abcam, GAPDH (Cat: 32 233) from Santa Cruz, and Wheat Germ Agglutinin Alexa Fluor 594 (Cat: W11262) from Invitrogen. NucBlue Live ReadyProbes Reagent (Invitrogen, Cat: R37605) was used to stain the nucleus for all live cell experiments and Hoechst 33258 (Invitrogen, H35569) was used to stain the nucleus for all fixed experiments. FLPEP (Cat: F1314) was purchased from Life Technologies; and Anti‐CD11b‐APC (Clone ICRF44, Cat: 301309), Anti‐CD45‐FITC (Clone HI30, Cat: 304005) isotype control mouse IgG1κ‐APC (Clone MOPC‐21, Cat: 400120) and Fc Receptor Blocking Solution (Cat: 422302) from Biolegend. Tubulin‐tracker Green (Invitrogen, Cat: T34075) was used according to the manufacturer's protocol. Detailed antibody information can be found in Tables S1 and S2 (Supporting Information). Poly(methacrylic acid) (PMA, MW = 100 000, Cat: 00578) and polyacrylamide (PAAm, MW = 5 000 000 – 6 000 000, Cat: 02806–50) were purchased from Polysciences. Poly(allylamine hydrochloride) (PAH, MW ≈200 000, Cat: 43092) was purchased from Alfa Aesar. 1,2‐dioleoyl‐3‐trimethylammonium‐propane (chloride salt) (DOTAP) was purchased from Avanti Polar Lipids (Cat: 890890C).

### Primary Neutrophil Isolation

All human subjects work was performed with approval of the University of Maryland, Baltimore Institutional Review Board (protocol number HP‐00107007). Informed consent was obtained from healthy adult human volunteers, and blood was drawn from peripheral veins and collected in tubes containing EDTA. Human neutrophils were isolated from the blood using MACSxpress Whole Blood Neutrophil Isolation kit (Miltenyi Biotec, Cat: 130‐104‐434) according to the manufacturer's protocol. Cells were either untreated (control), treated with Vinorelbine (10 µm) or Tetracaine (250 µm) and tethered onto TetherChips for 1 h at 37 °C. Anti‐CD45‐FITC (Clone HI30, Biolegend, Cat: 304005, 1:100) and Anti‐CD11b‐647 (Clone M1/70, Biolegend, Cat: 101218, 1:100) antibodies were used to confirm neutrophil presence. Tubulin tracker (Invitrogen Cat: T34075, 1:100) was used according to the manufacturer's protocol to confirm tubulin presence. McTN counts were based on live cell phase contrast images. McTNs were scored in a population of 100 cells/channel as previously described^[^ ^16^ ^]^ on a Nikon Ti2‐E inverted microscope at 60x magnification in phase contrast.

### Cell Culture and Differentiation

HL‐60 cells (ATCC, Cat: CCL‐240) were obtained as a gift from the Dr. Xuefang Cao lab at University of Maryland, Baltimore. HL‐60 cells were cultured in suspension in T‐75 tissue culture treated flasks (Sarstedt, Cat: 83‐3911‐002) with Iscove's Modified Dulbecco's Medium (IMDM, Invitrogen, Cat: 12440061) supplemented with 20% fetal bovine serum (R&D Systems, Cat: S11150H) and 1% penicillin‐streptomycin (Gemini, Cat: 400‐109) solution. MDA‐MB‐231TD (tumor‐derived) cells were produced in our lab by injecting MDA‐MB‐231 (ATCC, Cat: HTB‐26) cells stably expressing GFP and luciferase into mice and grown for 35 days. The tumors were resected, homogenized and the collected cells were used for experiments (described in more detail here^[^ ^23^ ^]^). MDA‐MB‐231TDs were grown in Dulbecco's Modified Eagle Medium (DMEM, Corning 10‐017‐CV), 10% FBS, and 1% penicillin‐streptomycin. All cells were maintained in a humidified environment at 37 °C and 5% CO_2_. All cells were also authenticated by the University of Maryland, Baltimore Biopolymer‐Genomics Core Laboratory via STR profiling using the Promega Geneprint 10 system. These cells were also confirmed negative for mycoplasma contamination throughout these experiments. HL‐60 cell cultures were passaged three times per week, maintaining cell densities between 10^5^ and 10^6^ cells/mL. HL‐60 cells were differentiated into a neutrophil‐like state by culturing at an initial density of 3×10^5^ cells/mL using IMDM growth media supplemented with 1.3% DMSO (Sigma) for 7 days. Once transfected with Bcl‐2, cells were maintained in complete media with 0.5 µg mL^−1^ puromycin for all experiments undifferentiated or differentiated. For migration experiments, HL‐60 Bcl‐2 mCherry cells were used and maintained in complete media with 0.5 µg mL^−1^ puromycin and 100 µg mL^−1^ hygromycin. All differentiated neutrophils were used at day 7 of DMSO treatment unless stated otherwise.

### Lentivirus Production and Transduction

Overexpression of Bcl‐2 in HL‐60 cells was performed by lentiviral transduction using the Lenti‐X Packaging System (Clontech, Cat: 631275) according to the manufacturer's instructions. The pCDH‐puro‐Bcl2 plasmid (Addgene plasmid #46971)^[^ ^54^ ^]^ was added into supplied nanoparticle complexes for 10 min and applied to Lenti‐X 293T cells to produce virus. Media was changed after 24 h and viral supernatant was harvested after 48 h, filtered and used to infect cells at an approximate MOI of 100 along with 8 µg mL^−1^ Polybrene (Sigma, Cat: TR‐1003‐G). The plate was then immediately centrifuged for 90 min at 1000 rpm. Two days after infection, cells were selected with 0.5 µg mL puromycin (Invitrogen, Cat: A1113803) and maintained in puromycin‐containing media for all experiments. Overexpression of mCherry (Addgene plasmid #129440) on HL‐60 Bcl‐2 cells was performed the same way as described above with the exception of hygromycin (EMD Millipore, Cat: 400052‐50mL) selection at 100 µg mL^−1^.

### Trypan Blue Exclusion Assay

To measure cell viability, HL‐60 or HL‐60 Bcl‐2 cells were counted daily from day 0 to day 14 of differentiation into neutrophils, using the trypan blue dye exclusion assay. Viability was calculated on a Countess II FL Automated Cell Counter (Thermo Fisher, Cat: AMQAX1000).

### Immunolabeling and Flow Cytometry

Differentiation of HL‐60 Bcl‐2 cells into a neutrophil‐like state was assessed, as previously described,^[^ ^38^ ^]^ by measuring the levels of CD11b and the formyl peptide receptor, FPR1, on the cell surface. Relative levels of FPR1 were assessed by binding of FLPEP (N‐formyl‐norleucyl‐leucyl‐phenylalanyl‐norleucyl‐tyrosyl‐lysine‐fluorscein (Life Technologies, Cat: F1314), a fluorescent ligand of the receptor. For detection of CD11b and FLPEP, cells were harvested, washed with phosphate‐buffered saline (PBS), and stained with CD11b‐APC (Clone ICRF44, Biolegend, Cat: 301310, 1:100) along with Fc Receptor Blocking Solution (Biolegend, Cat: 422302) for 45 min at 4 °C in FACS buffer (0.5% bovine serum albumin in PBS). After incubation, the samples were centrifuged, washed with cold PBS, and resuspended in FACS buffer. FLPEP was then added to a final concentration of 10 nm, incubated on ice for 10 min, and acquired with the BD FACS Canto II flow cytometer. FACS data was analyzed with FCS Express (De Novo Software). To determine the percentage of cells expressing CD11b and FLPEP, thresholds were set using a negative control for CD11b, isotype control mouse IgG1κ‐APC (Biolegend, Cat: 400120), and a non‐stained sample for FLPEP (Figure S3, Supporting Information). Detailed antibody information can be found in Table S2 (Supporting Information).

### RNA Library Preparation

HL‐60 Bcl‐2 cells were differentiated using 1.3% DMSO for 7 days and total RNA was extracted from day 0, 2, 4 and 7 of differentiation using the RNeasy Plant Mini kit (Qiagen, Cat: 74904) in biological triplicates. RNA quantity and quality were measured using a Nanodrop (ThermoFisher).

### RNA Sequencing and RNASeq Data Analysis

RNA was sent in biological triplicates for paired‐end bulk RNA sequencing at UMSOM IGS, including library preparation, QC, sequencing, and processing. Reads were aligned to the human genome (GRCh38.108) using HISAT2.^[^ ^55^ ^]^ The number of reads that aligned to the predicted coding regions was determined using HTSeq.^[^ ^56^ ^]^ Differentially expressed genes were identified by the DESeq2 Bioconductor package.^[^ ^57^ ^]^ Genes showing significant altered expression were defined using a False Discover rate (FDR) cutoff of FDR < 0.05 and a log2 fold change > ± 1. Supervised hierarchal clustering of differentially expressed neutrophil‐related genes were plotted in a heatmap using the pheatmap package.^[^ ^58^ ^]^

### Polyelectrolyte Multilayer (PEM) and Lipid Film Deposition

Multilayer film deposition was performed similar to methods previously published.^[^ ^29^ ^]^ Briefly, uncoated microfluidic slides (µ‐Slide VI 0.4 or µ‐Slide I Luer 0.8) (Ibidi, Cat: 80601 or 80191) were coated first with polycationic solution (PAH) for 15 min to introduce a primer/adhesion layer, then rinsed twice using pH 3.0 deionized (DI) water. The primer layer was followed by the addition of polyanionic PMA for 5 min and rinsed twice with pH 3.0 DI water. Then, nonionic PAAm was deposited and rinsed as described above. To thermally crosslink, microfluidic slides were placed in an oven at 90 °C for 8 h before the addition of the lipid. The next day, lipid was added to the microchannel for 5 min followed by two rinse steps. Following deposition, slides were allowed to air dry for 1 h at room temperature before use or stored in a desiccator until use.

### McTN Counting and Perimeter Tracing

HL‐60 Bcl‐2 cells were differentiated into neutrophils over 7 days, counted, and seeded onto a TetherChip microfluidic slide at 50 000 cells/well (Ibidi µ‐Slide I Luer 0.8, Cat: 80191). Cells were incubated for 45 min to allow for tethering. After 45 min, 3.7% formaldehyde/PBS was washed through each channel to fix cells for 10 min. Wheat germ agglutinin (WGA, Alexa Fluor 594 Conjugate, Invitrogen, Cat: W11262) was then added to each channel at a final concentration of 1:100 to visualize the cell membrane and Hoechst 33258 (1:1000) was used to stain the nucleus. McTNs were scored in a population of 100 cells/channel as previously described^[^ ^16^ ^]^ on a Nikon Ti2‐E inverted microscope at 60x magnification. Images for perimeter traces were acquired on an Olympus IX81 microscope with a Fluoview FV1000 confocal laser scanning system at 60x magnification. Perimeter image analysis was quantified on ImageJ with Fiji as previously shown^[^ ^29^ ^]^ and used as a correlative parameter for McTNs with a higher perimeter associating with a greater McTN positivity.

### Surface Marker Staining

HL‐60 Bcl‐2 cells were differentiated into neutrophils for 7 days, stained with CD11b (Clone M1/70, Biolegend, Cat: 101218, 1:100) and tethered onto TetherChips for 45 min. Cells were then fixed with 4% formaldehyde in PBS for 10 min and then stained with WGA (Alexa Fluor 488 or 594 conjugate, Invitrogen, 1:100) overnight. The next day cells were washed with PBS and mounted with Fluoromount‐G (Invitrogen, Cat: 00‐4958‐02).

### Immunofluorescence

For intracellular staining, undifferentiated HL‐60 Bcl‐2 cells and day 7 differentiated HL‐60 Bcl‐2(N) cells were allowed to tether for 45 min onto a TetherChip microfluidic slide (µ‐Slide I Luer 0.8) then fixed with 3.7% formaldehyde diluted in PBS, washed and stained with WGA (Alexa Fluor 647 conjugate, Invitrogen, 1:100) for 15 min. Cells were then permeabilized in 0.1% Triton‐X 100 diluted in PBS, blocked in 5% bovine serum albumin (BSA) diluted in PBS, and incubated overnight at 4 °C in 5% BSA/PBS with an antibody against detyrosinated α‐tubulin (1:10000, RevMAb Clone RM444, Cat: 31‐1335‐00), acetylated α‐tubulin (1:1000, Cell Signaling Technologies, Cat: 5335S), or vimentin (EPR3776) (1:1000, Abcam, Cat: ab92547) and α‐tubulin (1:1000, Sigma‐Aldrich, Cat: T6199). Secondary antibodies, anti‐mouse Alexa Fluor 488 (1:1000, Invitrogen, Cat: A‐11001), anti‐rabbit Alexa Fluor 594 (1:1000, Invitrogen, Cat: A11012), and Hoechst 33258 (1:1000, Invitrogen, Cat: H1398) were diluted in BSA/PBS, added to each channel, and incubated for 2 h at room temperature. Finally, channels were washed with PBS and mounted with Fluoromount‐G (Invitrogen, Cat: 00‐4958‐02). Images were acquired using an Olympus IX81 microscope with a Fluoview FV1000 confocal laser scanning system or a Nikon Ti2‐E inverted microscope with a Nikon AX‐R confocal system with identical exposure and laser settings in each experiment replicate. Detailed antibody information can be found in Table S1 (Supporting Information).

### Immunoblotting

Confluent flasks of HL‐60 Bcl‐2 cells differentiated with 1.3% DMSO over 7 days, were collected at each day, spun down for 5 min at 1000 rpm, washed with PBS, and lysed in RIPA buffer containing 0.1% phosphatase inhibitor and 1% protease inhibitor on ice. Lysates were centrifuged at 14000 rpm for 20 min at 4 °C and protein concentration was measured using the BioRad DC protein assay (Cat: 5000112) according to manufacturer's instructions. Total protein (30 µg) was separated by SDS‐PAGE on NuPage 4–12% Bis‐Tris protein gels (Invitrogen, Cat: NP0335BOX) and then transferred to PVDF membranes using the eBlot L1 Fast Wet Transfer System (Genscript, Cat: L00727). Membranes were blocked in 5% BSA in TBS with 0.1% Tween‐20 (TBS‐T) for 1 h at room temperature followed by an overnight incubation at 4 °C in Bcl‐2 (1:1000), detyrosinated α‐tubulin (1:1000), acetylated α‐tubulin (lys40) (1:1000), total α‐tubulin (1:1000), vimentin (1:1000) or GAPDH (1:5000) antibody in 5% BSA diluted in TBS‐T. Secondary antibodies to IgG conjugated to horseradish peroxidase were used (1:5000, Cat: 711‐035‐152 and 715‐035‐150, Jackson ImmunoResearch Laboratories) and visualized using ECL chemiluminescence. Blots were imaged on the iBright Imaging System (Thermo Fisher) using the smart exposure feature and densitometry analysis was performed in ImageJ and graphed in GraphPad Prism. Detailed antibody information can be found in Table S1 (Supporting Information).

### Drug Treatments

Vinorelbine tartrate (BioVision, Cat: 1957‐5) was resuspended in PBS and used at a final concentration of 10 µm. Paclitaxel (Enzo, Cat: BML‐T104‐0005) was resuspended in DMSO and used at a final concentration of 1 µm. Tetracaine hydrochloride (Sigma, Cat: T7508‐5g) was resuspended in ddH_2_O and used at a final concentration of 250 µm. Cytochalasin D (Sigma, Cat: C8273‐1mg) was resuspended in DMSO and used at a final concentration of 5 µm. For McTN formation assays, day 7 differentiated neutrophils were treated and tethered onto a TetherChip with the indicated drug for 1 h, fixed, stained with Hoechst and WGA594, mounted with Fluoromount‐G, and imaged for analysis. For immunoblotting, undifferentiated HL‐60 cells or day 7 differentiated neutrophils were treated with Vinorelbine for 1 h and then lysates were collected to analyze changes in α‐tubulin post‐translational modifications. For longer‐term assays such as clustering, migration, and attachment, day 7 differentiated neutrophils were treated with Vinorelbine for 1 h and then also plated in Vinorelbine‐containing media since Vinorelbine is a reversible drug. Detailed information on each drug can be found in Table S3 (Supporting Information).

### CellTiter‐Glo Viability Assay

HL‐60 Bcl‐2 cells were plated at 5000 cells/well in 100 µL in a 96‐well and treated with the indicated concentrations of Vinorelbine and 1 µm Staurosporine for the indicated time points. To assess viability, CellTiter‐Glo (Promega, Cat: G7571) was used according to the manufacturer's instructions. Briefly, at the indicated time points, 25 µL of 1:1 mixed reagent was added to each well and the plate was covered with aluminum foil and rocked gently at room temperature for 10 min to allow lysis. Luminescence was then read on a plate reader and values were normalized to t = 0.

### xCELLigence Attachment and Migration for Differentiated Neutrophils

Real‐time dynamic monitoring of cellular reattachment from suspension as well as migration were measured using the xCELLigence RTCA DP analyzer (Agilent). For reattachment assays, E‐plates, electronic microplates with biosensors on the bottom surface, were used. The inside of the wells were coated with fibronectin (Sigma, Cat: F4759) diluted in PBS at 50 µg mL^−1^ overnight at 4 °C. Prior to cell seeding, wells were washed with PBS three times. Day 7 differentiated neutrophils were then seeded at 200 000 cells/well in 100 µL for each condition described.

For migration assays, CIM plates, which are analogous to an electronically integrated Boyden chamber, were utilized (Figure 8A). A CIM‐plate consists of two separate parts: an upper chamber with a microporous membrane embedded with gold microelectrodes on its underside and a lower chamber. Fibronectin was coated on the underside of the top chamber of each CIM plate at 50 µg mL^−1^ in PBS overnight at 4 °C and was then washed with PBS three times just before cell seeding, as previously shown.^[^ ^59^ ^]^ fMLP (formyl‐Met‐Leu‐Phe), a chemoattractant for neutrophils, was diluted to a concentration of 200 nm in complete media and added to the lower chamber. Chambers were attached together, and day 7 differentiated neutrophils were seeded at 200 000 cells/well in 100 µL to the upper chamber in media without chemoattractant. Attachment and migration are both expressed as a change in cell index, an arbitrary unit reflecting the relative change in electrical impedance from cell‐electrode interaction across microelectronic sensor arrays. Impedance for both migration and reattachment were measured every 5 min for 8 h. Upon completion of the experiment, the cartridges were taken apart and the membranes were fixed, stained with Hoechst (1:1000) and WGA (1:100) for 15 min and then a glass coverslip was mounted on top for imaging.

### Time‐Lapse Microscopy

Undifferentiated HL‐60 cells and day 7 differentiated neutrophils were either untreated or treated with PBS (vehicle) or 10 µm Vinorelbine and seeded over fibronectin coated 24‐well plates. Fibronectin was coated in each well at 50 µg mL^−1^ in PBS overnight at 4 °C and was then washed with PBS three times just before cell seeding. Cells were seeded at 50 000 cells/well either in media, media containing PBS or media containing 10 µm Vinorelbine and phase contrast images of cells reattaching were captured every 2 min for 24 h. The images shown are at the 1‐h time point since most of the cells had reattached by then. Images were taken at 10x on a Nikon Ti2‐E inverted microscope with a Tokai Hit stage top incubator.

### Clustering Assay and Image Analysis

HL‐60 Bcl‐2 cells were differentiated for 7 days, counted, and plated in low‐attach 96‐well plates (Corning, Cat: 3474) at 40 000 cells per well. Cells were allowed to cluster for 24 h and then collected and tethered onto a TetherChip microfluidic slide (Ibidi µ‐Slide VI 0.4, Cat: 80601) for 30 min at 37 °C. Cells were subsequently fixed using 4% formaldehyde and stained with Hoechst 33258 (1:1000). Whole channel scanned images were acquired and stitched using the Nikon Ti2‐E inverted microscope at 4x magnification. Image analysis was done in ImageJ with Fiji by automatic thresholding and subsequently analyzing particles to quantify cluster number and size (area). Objects smaller than 75 µm^2^ (smaller than the size of a single nucleus) were considered cellular debris and excluded from the analysis. From the binarized thresholded images, a measured clustering efficiency metric is reported by comparing the number of individual clusters over time. Individual values at t = 0 were divided by respective experimental final cluster numbers (t = 24 h) for each condition and reported as clustering efficiency.

### Co‐Culture Clustering Assays

For all co‐culture clustering assays, 40 000 MDA‐MB‐231TD^[^ ^23^ ^]^ cells (tumor cells, GFP‐labeled) and 40 000 HL‐60 Bcl‐2(N) cells (day 7 differentiated neutrophils) stained with Anti‐CD11b‐APC (Clone M1/70, Biolegend, Cat: 101218, 1:100) were allowed to co‐culture in low‐attach plates (Corning, Cat: 3474) either in vehicle control (PBS) or 10 µm Vinorelbine for 6 h before seeding onto a TetherChip microfluidic slide (Ibidi µ‐Slide VI 0.4) for 30 min at 37 °C. Cells were subsequently fixed using 4% formaldehyde and stained with Hoechst 33258 (1:1000). Whole channel scanned images were acquired and stitched using the Nikon Ti2‐E inverted microscope at 4x magnification and image analysis for clustering efficiency and average cluster size was done as described above in the clustering assay section.

### Co‐Culture Migration Assays

For all co‐culture migration assays, MDA‐MB‐231TD^[^ ^23^ ^]^ cells (tumor cells) and HL‐60 Bcl‐2(N) cells transfected with a mCherry lentivirus (day 7 differentiated neutrophils) were allowed to co‐culture overnight before seeding onto migration cartridges. Real‐time dynamic monitoring of co‐culture migration was measured using the xCELLigence RTCA DP analyzer (Agilent, Figure 8A), as described above. FBS, a chemoattractant for tumor cells, in basal DMEM media was added to the lower chamber. The two chambers were attached together (without fibronectin coating) and either 40 000 MDA‐MB‐231TD cells alone; 40 000 HL‐60 Bcl‐2(N) cells alone, 40 000 undifferentiated HL‐60 Bcl‐2 cells alone, a 1:1 ratio (40 000 MDA‐MB‐231TD: 40 000 HL‐60 Bcl‐2(N)) of tumor cells to differentiated neutrophils or a 1:1 ratio (40 000 MDA‐MB‐231TD: 40 000 HL‐60 Bcl‐2) of tumor cells to undifferentiated HL‐60 cells were seeded in 100 µL to the upper chamber in media without FBS. Impedance was measured every 5 min for 24 h. For experiments using 200 nm fMLP as the chemoattractant for neutrophils, fibronectin was coated on the underside of the top chamber of each CIM plate at 50 µg mL^−1^ overnight at 4 °C and was then washed with PBS three times just before cell seeding, as previously described.^[^ ^59^ ^]^ 40 000 MDA‐MB‐231TD cells alone, 200 000 undifferentiated HL‐60 Bcl‐2 cells alone, 200 000 HL‐60 Bcl‐2(N) cells alone, or a 5:1 ratio (40 000 MDA‐MB‐231TD: 200 000 HL‐60 Bcl‐2) of tumor cells to undifferentiated HL‐60 cells or a 5:1 ratio (40 000 MDA‐MB‐231TD: 200 000 HL‐60 Bcl‐2(N)) of tumor cells to differentiated neutrophils were seeded in 100 µL to the upper chamber in media without fMLP. Impedance was measured every 5 min for 8 h. Upon completion of the experiment, the cartridges were taken apart and the membranes were fixed, stained with Hoechst (1:1000) for 15 min and then a glass coverslip was mounted on top for imaging (Figure 8A).

### Statistical Analysis

GraphPad Prism (version 9/10) was used to perform all statistical comparisons. One‐way and two‐way ANOVA tests were performed with a Bonferroni multiple comparisons post‐test as indicated. Two‐tailed t‐tests were performed when indicated. A p‐value of 0.05 or less was considered statistically significant.

## Conflict of Interest

The University of Maryland School of Medicine owns patents on the subject of microtentacles and microfluidic cell tethering that list one of the authors on the current manuscript (Stuart S. Martin) as an inventor.

## Author Contributions

The conceptualization and design of the study were carried out by J.A.J. and S.S.M. Data acquisition was conducted by J.A.J., while analysis and interpretation involved contributions from J.A.J., K.N.T., M.I.V., D.A.A., A.M., M.L.M., D.E.G., K.T.C., M.B.S., M.J.N., and S.S.M. J.A.J. was responsible for drafting the manuscript, with edits made by J.A.J. along with K.N.T., M.I.V., D.A.A., A.M., M.L.M., D.E.G., K.T.C., M.B.S., M.J.N., and S.S.M. All authors read and approved the final manuscript.

## Supporting information



Supporting Information

Supplemental Video 1

Supplemental Video 2

Supplemental Video 3

Supplemental Video 4

## Data Availability

The data that support the findings of this study are available from the corresponding author upon reasonable request.
