# Supplementary material for: Tubulin‐Based Microtentacles Aid in Heterotypic Clustering of Neutrophil‐Differentiated HL‐60 Cells and Breast Tumor Cells
Source: Adv Sci (Weinh). 2024 Dec 18;12(6):2409260. doi: 10.1002/advs.202409260 (PMC11809343; doi:10.1002/advs.202409260)
Supplement: Supplementary file 1 — Supporting Information [file ADVS-12-2409260-s002.docx]

**Supplemental Files:**

**
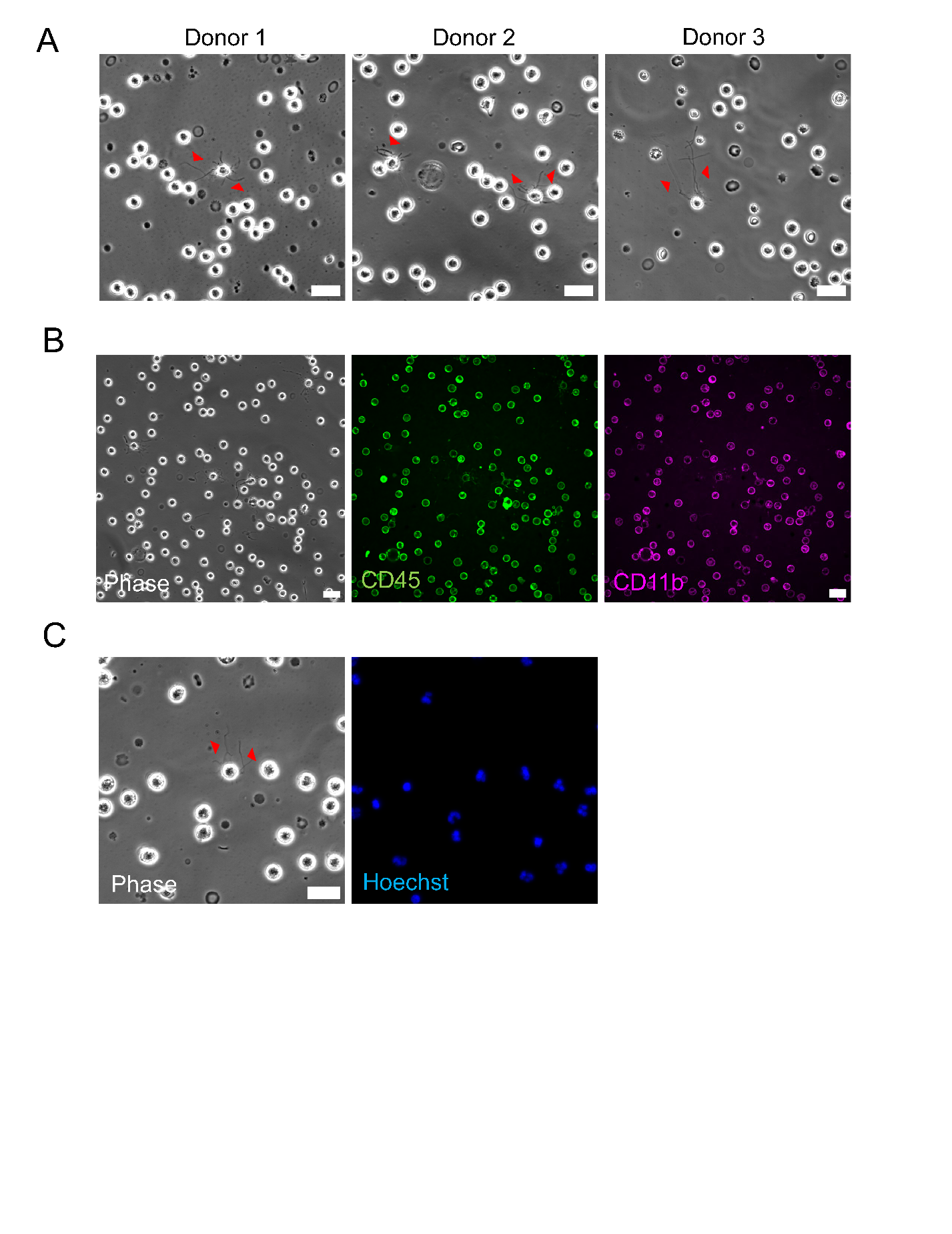
**

**Figure S1: Primary human neutrophils produce tubulin-based protrusions. A.** Representative population phase contrast images of live isolated primary human neutrophils from three separate human donors. Scale bar = 20µm. **B.** Representative population phase contrast (left) scale bar = 20μm, CD45-stained (middle) and CD11b-stained (right) live primary neutrophils illustrating the cells that were isolated are neutrophils. Scale bar = 20μm. **C.** Representative population phase contrast (left) and Hoechst-stained (right) live primary neutrophils displaying

that microtentacles are not made of DNA. Scale bar = 20μm. Red arrows indicate microtentacles.

**
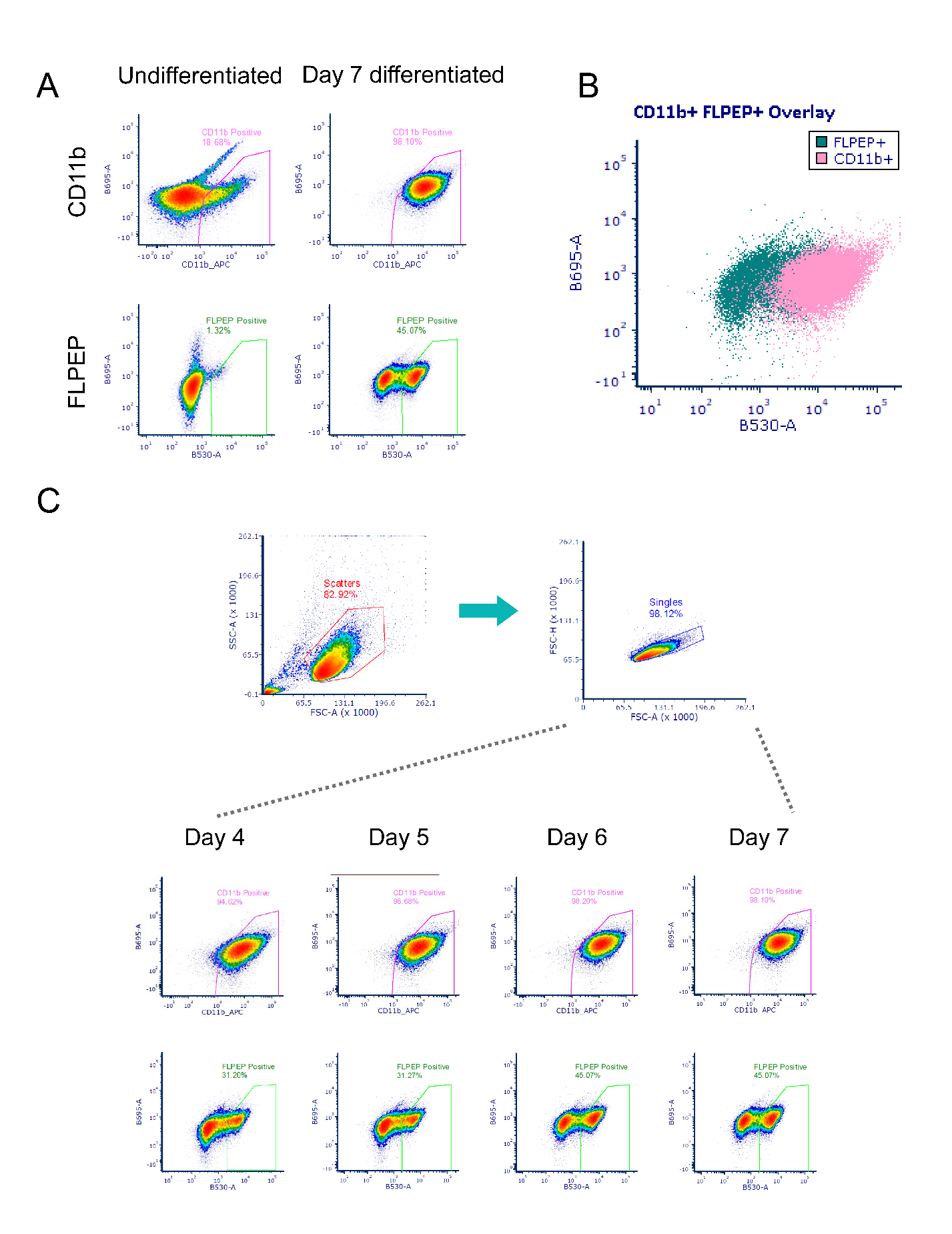
**

**Figure S2: Confirmation of HL-60 Bcl-2 differentiation into neutrophils with 1.3% DMSO. A.** Density-colored scatter plots of expression values for CD11b and FLPEP for undifferentiated HL-60 Bcl-2 cells versus HL-60 Bcl-2 cells differentiated into neutrophil-like cells after 7 days of 1.3% DMSO treatment. **B.** Density-colored scatter plot of expression values of singlets for both CD11b and FLPEP overlapped for HL-60 Bcl-2 cells differentiated into neutrophil-like cells after 7 days of 1.3% DMSO treatment. **C.** Representative density-colored scatter plots for CD11b and FLPEP-positive differentiated HL-60 Bcl-2 cells from 4 to 7 days of 1.3% DMSO treatment.

**Figure S3: Flow cytometry analysis of HL-60 Bcl-2 differentiation into neutrophils. A-F.** Density-colored scatter plots of expression values for CD11b (left panel) and FLPEP (right panel) for undifferentiated HL-60 Bcl-2 cells versus HL-60 Bcl-2 cells differentiated into neutrophils after 4 days of DMSO treatment. A) Undifferentiated and unstained negative control sample. B) Undifferentiated isotype control sample. C) Undifferentiated sample. D) Day 4 differentiated into neutrophils unstained negative control sample. E) Day 4 differentiated into neutrophils isotype positive control sample. F) Day 4 differentiated neutrophil sample. **G-L.** Density-colored scatter plots of expression values for CD11b (left panel) and FLPEP (right panel) for undifferentiated HL-60 Bcl-2 cells versus HL-60 Bcl-2 cells differentiated into neutrophils after 7 days of DMSO treatment. G) Undifferentiated and unstained negative control sample. H) Undifferentiated isotype positive control sample. I) Undifferentiated sample. J) Day 7 differentiated neutrophils unstained negative control sample. K) Day 7 differentiated neutrophils isotype positive control sample. L) Day 7 differentiated neutrophil sample.


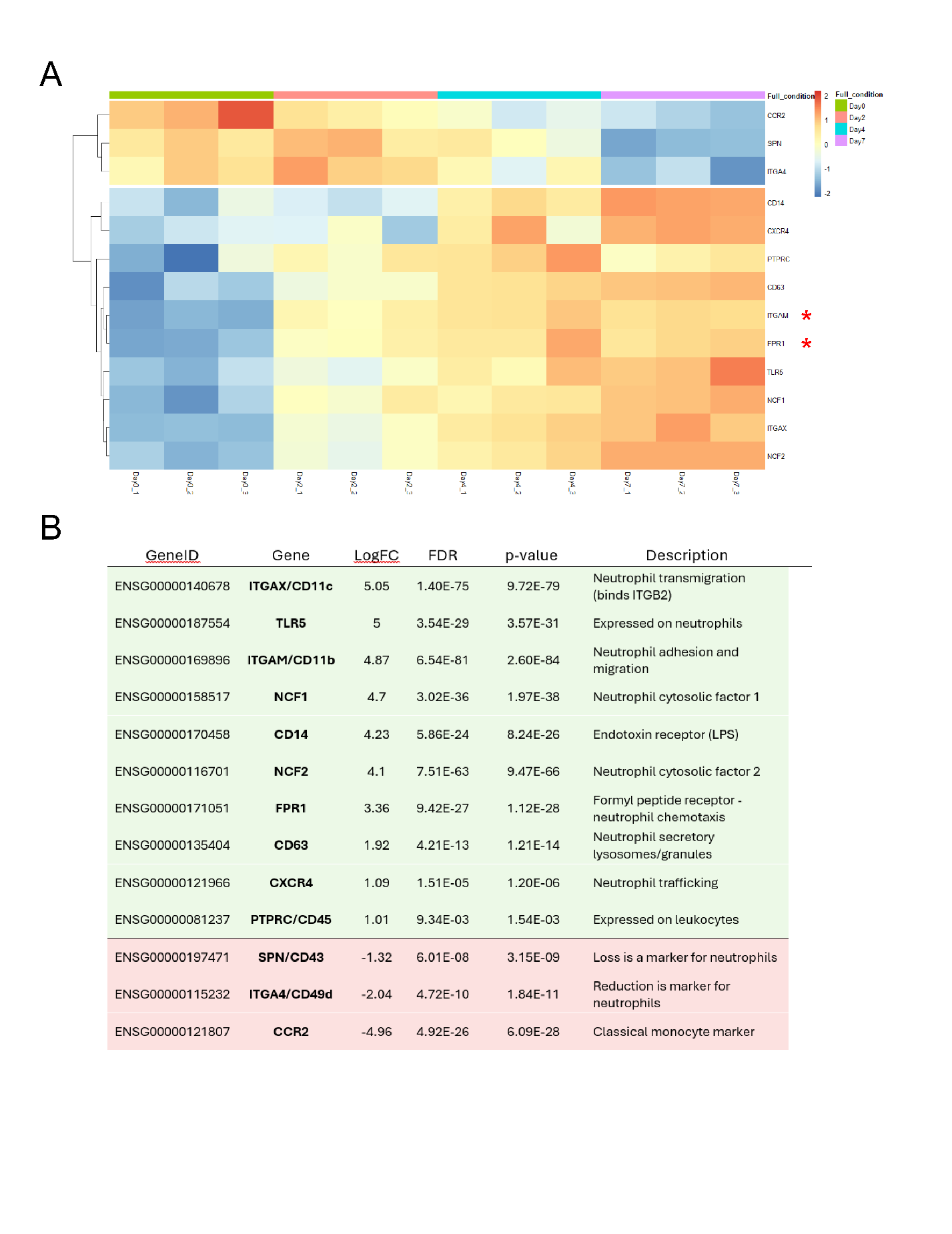


**Figure S4: RNASeq analysis of HL-60 Bcl-2 cells differentiated into neutrophils. A.** Heatmap of a list of differentially expressed genes (DEGs) associated with HL-60 Bcl-2 differentiation into neutrophils over 7 days with days 0, 2, 4 and 7 shown. Red indicates a high z-score while blue indicates a low z-score. ITGAM (CD11b) and FPR1 are starred because those are the markers used throughout the paper. Triplicates from each indicated time point are shown. **B.** Table of listed DEGs from the heatmap with GeneID, Log fold change (LogFC) values, false discovery rate (FDR) values, p-values and a short description of each gene. Green highlight indicates upregulation of gene while red highlight indicates downregulation of gene.

**
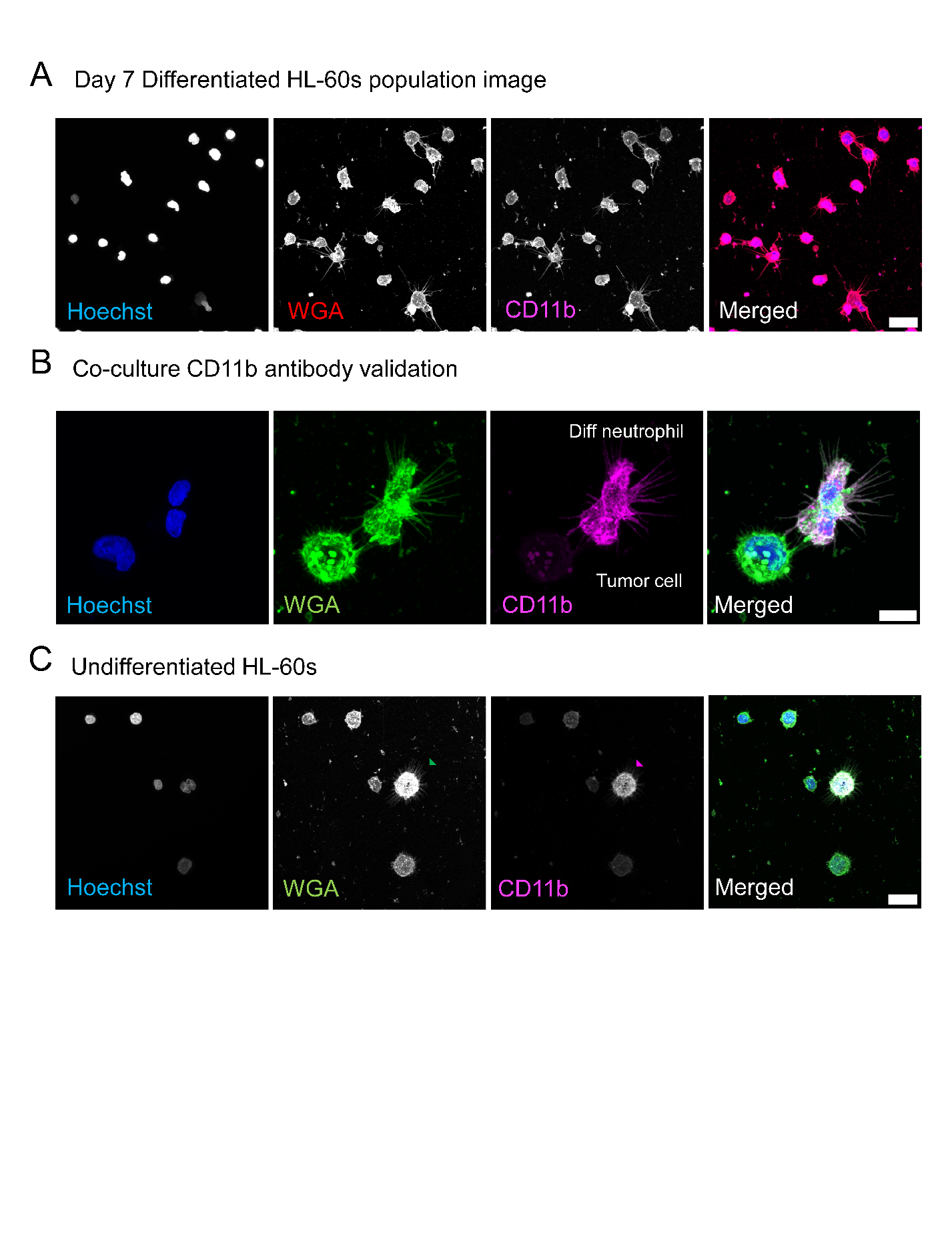
**

**Figure S5: Immunofluorescence confirmation of HL-60 Bcl-2 differentiation into neutrophils. A.** Representative population image of day 7 differentiated neutrophils tethered, fixed, and stained with Hoechst, WGA and CD11b. Scale bar = 20µm. **B.** Representative image of day 7 differentiated neutrophil – tumor cell co-culture tethered, fixed and stained with Hoechst, WGA and CD11b for CD11b antibody validation. **C.** Representative image of undifferentiated HL-60 Bcl-2 cells tethered, fixed and stained with Hoechst, WGA and CD11b confirming only the cell with McTNs is CD11b positive. Images were taken at 60x magnification using a Nikon Ti2-E inverted microscope with a Nikon AX-R confocal system. Images were denoised in a post-processing step using NIS Elements. Scale bar = 20µm.


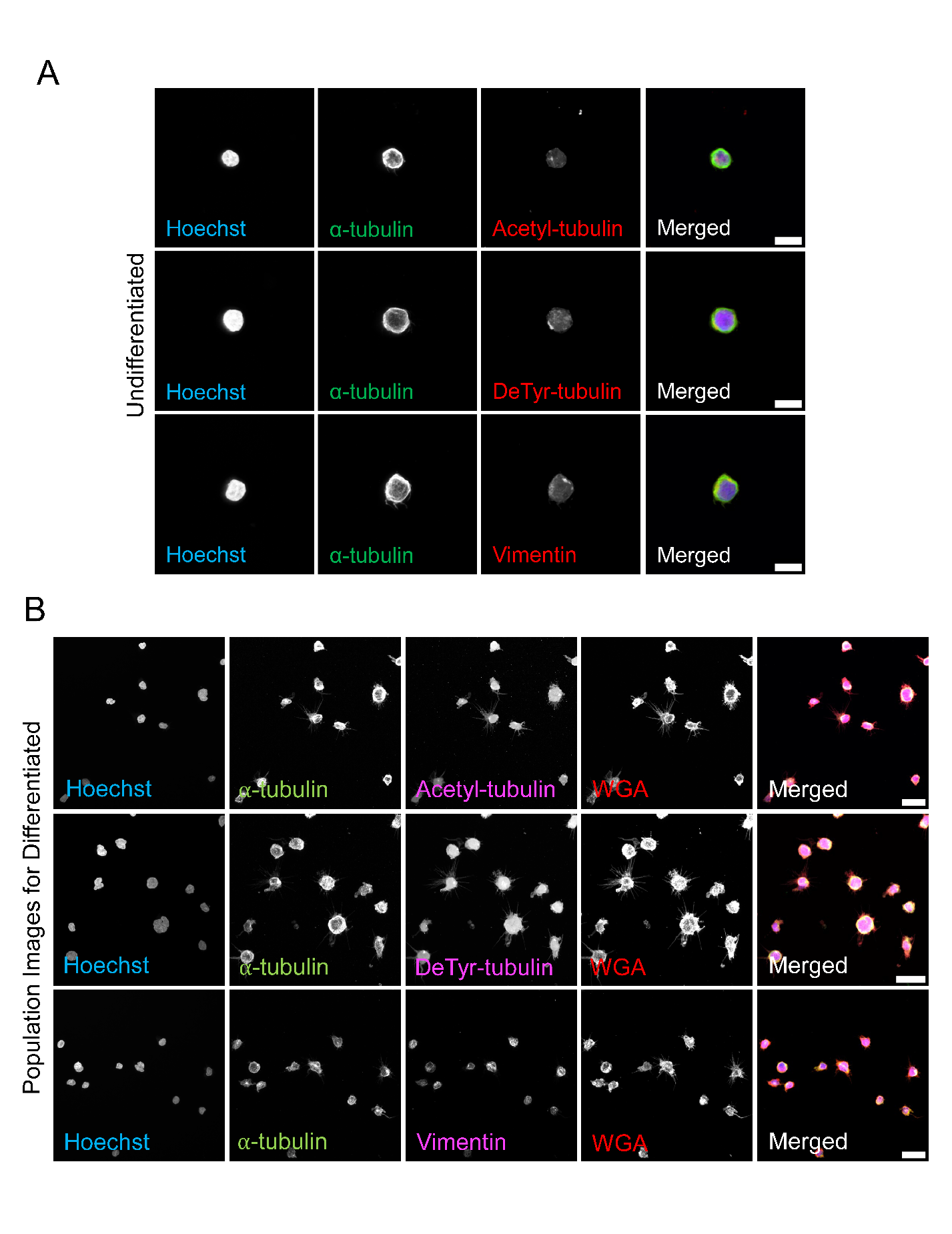


**Figure S6: Immunofluorescence of post-translational tubulin modifications in undifferentiated HL-60 Bcl-2 cells and differentiated neutrophils. A.** Immunofluorescence images of tethered and fixed HL-60 Bcl-2 undifferentiated cells stained with Hoechst, α-tubulin and either acetylated α-tubulin, detyrosinated α-tubulin or vimentin. Images were taken at 60x magnification using a Nikon Ti2-E inverted microscope with a Nikon AX-R confocal system. Images were denoised in a post-processing step using NIS Elements. Scale bar = 10µm. **B.** Representative population images of tethered and fixed day 7 differentiated neutrophils stained with Hoechst, α-tubulin, WGA and either acetylated α-tubulin, detyrosinated α-tubulin or vimentin. Images were taken at 60x magnification using a Nikon Ti2-E inverted microscope with a Nikon AX-R confocal system. Images were denoised in a post-processing step using NIS Elements. Scale bar = 20µm.


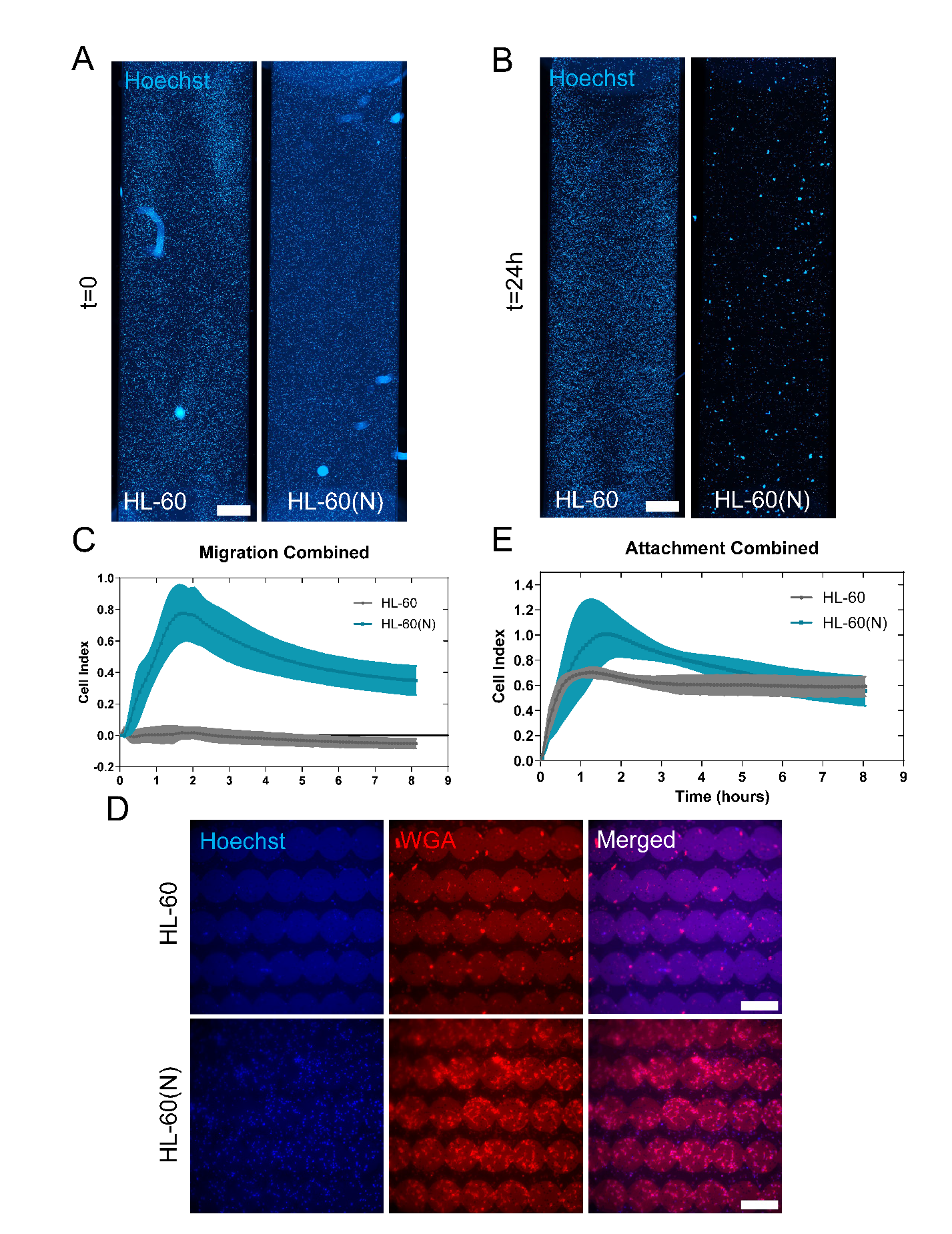


**Figure S7: Differentiation of HL-60 Bcl-2 cells into neutrophils induces cluster formation, migration, and attachment. A.** Full channel representative scans of undifferentiated HL-60 Bcl-2 cells (left panel) or day 7 differentiated neutrophils (right panel) tethered onto TetherChips stained with Hoechst at t=0 for clustering. Scale bar = 1000µm. **B.** Full channel representative scans of undifferentiated HL-60 Bcl-2 cells (left panel) or day 7 differentiated neutrophils (right panel) that were allowed to cluster for 24 hours and then tethered onto TetherChips stained with Hoechst. Scale bar = 1000µm. **C.** Graph of combined migration efficiency of all three biological replicates of undifferentiated versus day 7 differentiated HL-60 Bcl-2 cells towards fMLP over the course of 8 hours. Error bars indicate ± standard deviation, n=3. **D.** Fluorescent zoomed-out images of the membrane of fibronectin-coated xCelligence migration CIM cartridges that were formaldehyde-fixed and stained with Hoechst and WGA with undifferentiated HL-60 Bcl-2 cells versus day 7 differentiated neutrophils that migrated through pores towards 200nM fMLP. Images were taken at 40x magnification using a Nikon Ti2-E inverted microscope. Scale bar = 200µm. **E.** Graph of combined reattachment efficiency of all three biological replicates of undifferentiated HL-60 Bcl-2 cells versus day 7 differentiated neutrophils over the course of 8 hours. Error bars indicate ± standard deviation, n=3.


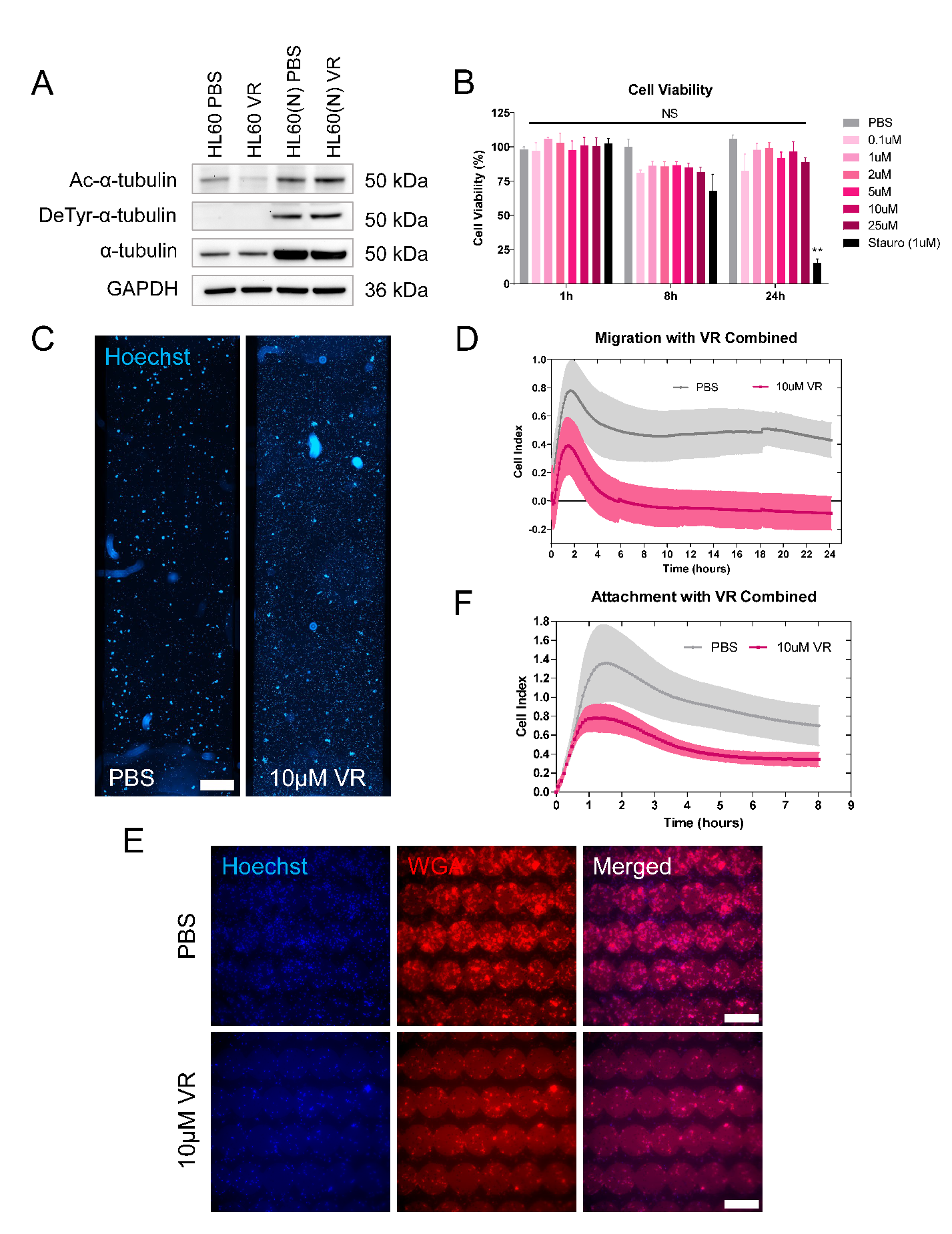


**Figure S8: Vinorelbine treatment disrupts cluster formation and decreases migration and reattachment of differentiated neutrophils. A.** Western blot analysis of undifferentiated HL-60 Bcl-2 cells versus day 7 differentiated neutrophils treated with PBS (control) or 10µM Vinorelbine and probed for acetylated α-tubulin, detyrosinated α-tubulin, α-tubulin and GAPDH. **B.** Luminescent cell viability assay using CellTiter-Glo with varying concentrations of Vinorelbine for 1h, 8h and 24h compared to Staurosporine (1µM) as a positive control of cell death. Cell viability % was calculated by dividing the luminescence (RLU) at each time point by the RLU at t=0 when the drug was first added and multiplying by 100. Data are shown as mean ± standard error of mean, n=3. **p < 0.01 versus 1h (Two-way ANOVA with Bonferroni post-test). **C.** Full channel representative scans of day 7 differentiated neutrophils treated with PBS (left panel, control) or 10µM Vinorelbine (right panel) that were allowed to cluster for 24 hours and then tethered onto TetherChips stained with Hoechst. Scale bar = 1000µm. **D.** Graph of combined migration efficiency of all three biological replicates of day 7 differentiated neutrophils treated with either PBS or 10µM Vinorelbine towards fMLP over the course of 24 hours. Error bars indicate ± standard deviation, n=3. **E.** Fluorescent zoomed-out images of the membranes of fibronectin-coated xCelligence migration CIM cartridges that were formaldehyde-fixed and stained with Hoechst and WGA with PBS (control) or Vinorelbine-treated day 7 differentiated neutrophils that migrated through pores towards 200nM fMLP. Images were taken at 40x magnification using a Nikon Ti2-E inverted microscope. Scale bar = 200µm. **F.** Graph of combined reattachment efficiency of all three biological replicates of day 7 differentiated neutrophils treated with either PBS (control) or 10µM Vinorelbine towards fMLP over the course of 8 hours. Error bars indicate ± standard deviation, n=3.


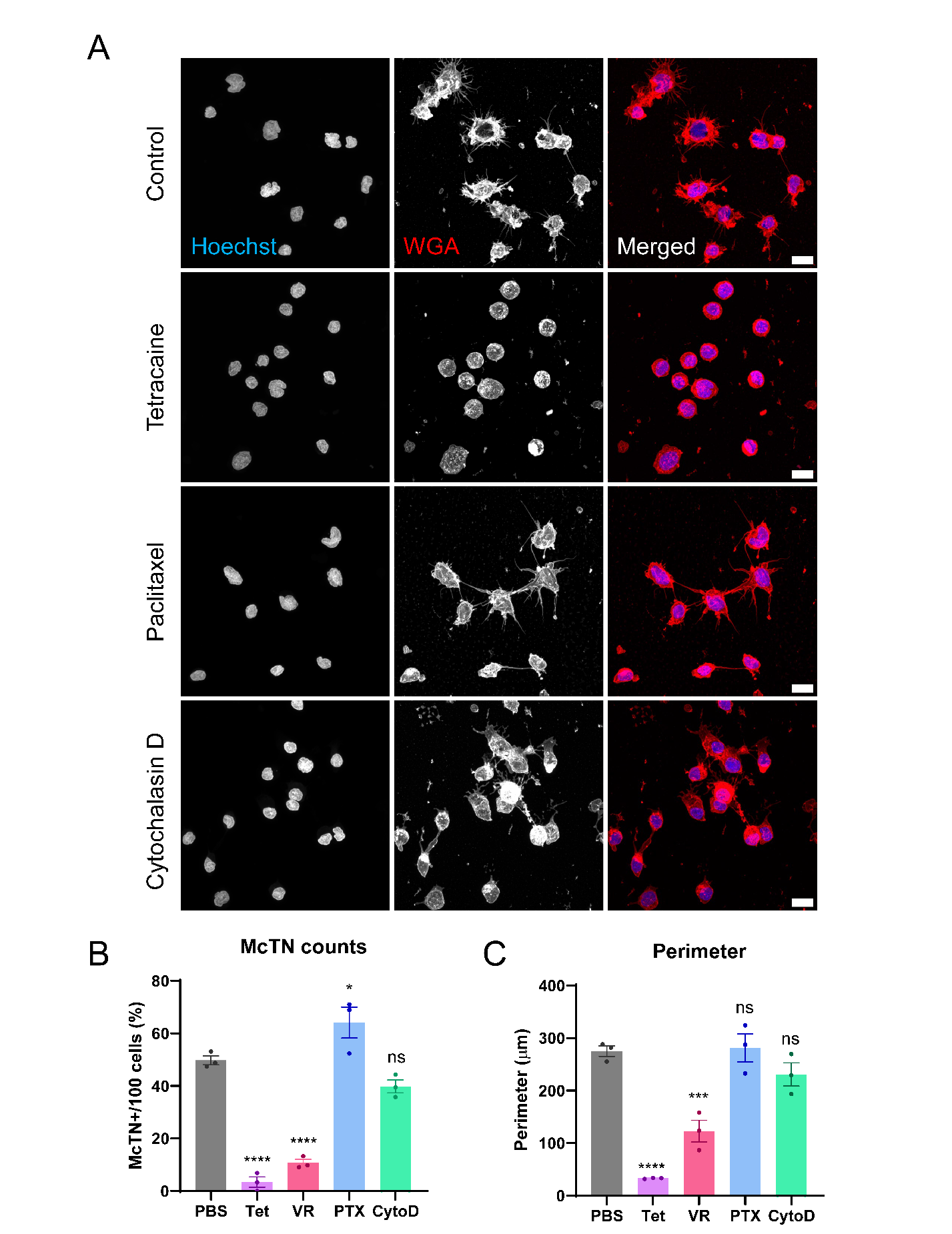


**Figure S9: Effects of tubulin-modulating drugs on differentiated neutrophils. A.** Representative images of vehicle (PBS), Tetracaine (250µM), Paclitaxel (1µM) or Cytochalasin D (5µM) - treated day 7 differentiated neutrophils that were tethered, fixed and stained with Hoechst and WGA. Scale bar = 10µm. **B.** McTN quantification of HL-60(N) cells treated with vehicle (PBS) versus 250µM Tetracaine, 10µM Vinorelbine, 1µM Paclitaxel or 5µM Cytochalasin D tethered on TetherChips. Data represents quantification of McTN frequency from three independent experiments with at least 100 cells counted for each. Data are shown as mean ± standard error of mean, n=3. *p < 0.05, ****p < 0.0001 versus PBS (One-way ANOVA with Bonferroni post-test). **C.** Quantification of the perimeter of day 7 differentiated neutrophils treated with vehicle (PBS) versus Tetracaine, Vinorelbine, Paclitaxel or Cytochalasin D analyzed by ImageJ. Data are shown as mean ± standard error of mean, n=3. ***p < 0.001, ****p < 0.0001 versus PBS (One-way ANOVA with Bonferroni post-test).

**
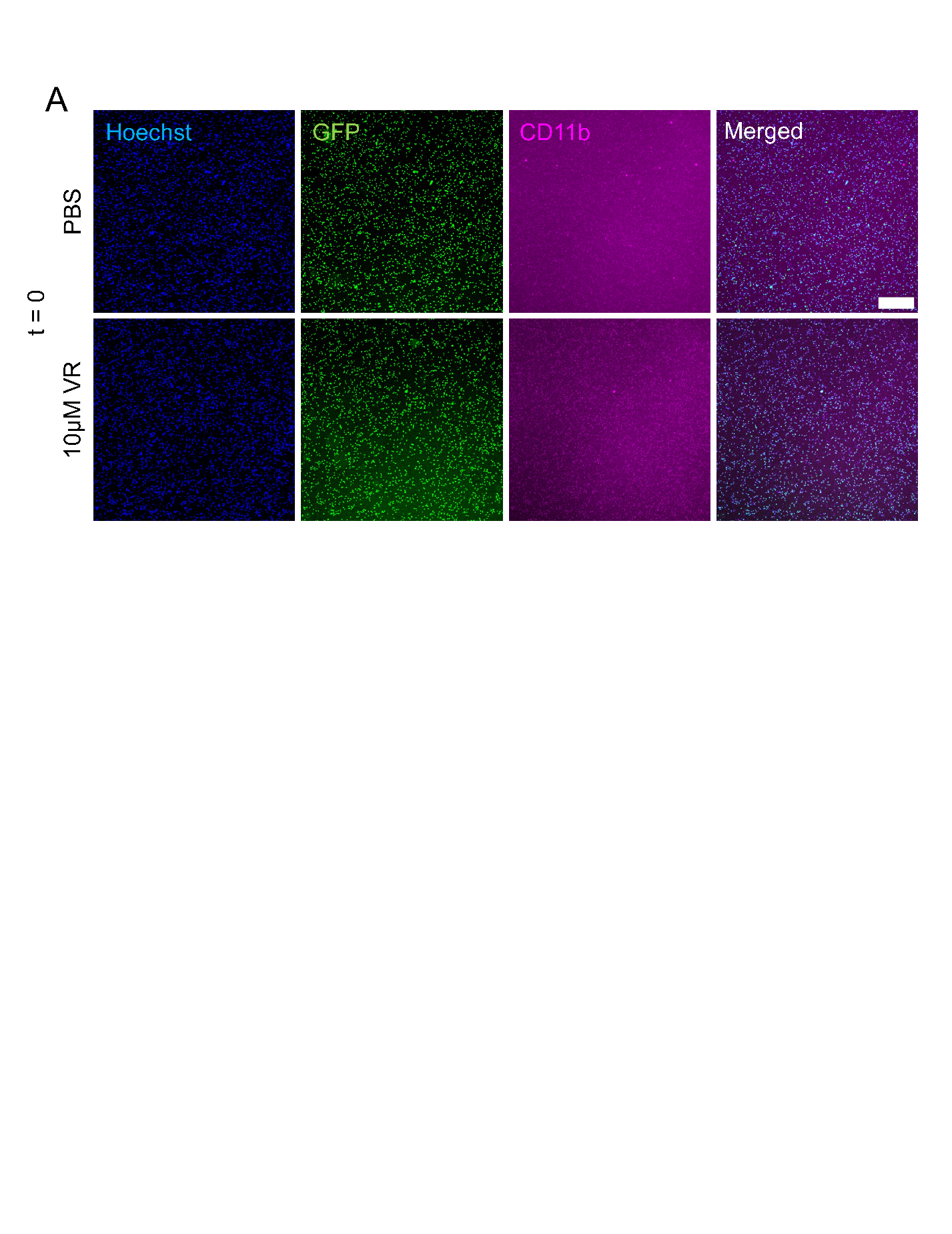
**

**Figure S10: Differentiated neutrophils and tumor cells form heterotypic clusters that can be inhibited with Vinorelbine. A.** Representative images of t=0 of heterotypic clustering between GFP-labeled MDA-MB-231TD tumor cells and CD11b stained day 7 differentiated neutrophils that were treated with PBS or 10µM Vinorelbine, tethered and fixed. Images were taken at 4x magnification using a Nikon Ti2-E inverted microscope. Scale bar = 500µm.


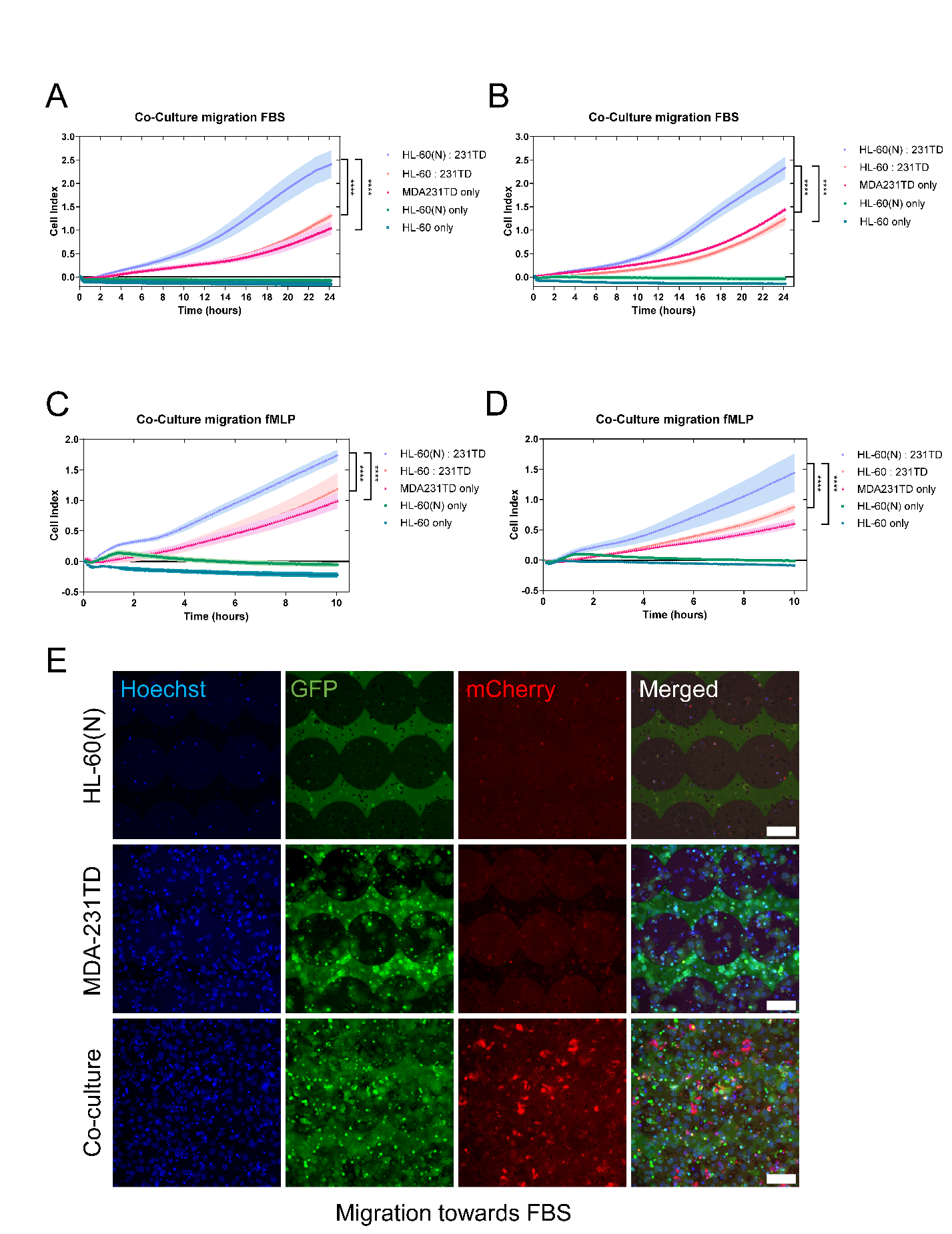


**Figure S11: Co-culturing day 7 differentiated neutrophils with tumor cells enhances migration. A-B.** Graph of the other two experimental replicates for migration efficiency of undifferentiated HL-60 Bcl-2 cells only, differentiated neutrophils only, MDA-MB-231TD cells only, a 1:1 co-culture of undifferentiated HL-60 Bcl-2 cells and MDA-MB-231TD cells, and a 1:1 co-culture of differentiated neutrophils and MDA-MB-231TD cells towards FBS (tumor cell attractant) over the course of 24 hours. Error bars indicate mean ± SD, each experiment was performed in triplicates. A) ****, p < 0.0001 of differentiated neutrophils : tumor cells co-culture versus tumor cells only from 10.5 hours to 24 hours and undifferentiated HL-60 Bcl-2 cells : tumor cells co-culture versus differentiated neutrophil : tumor cells co-culture from 10.5 hours to 24 hours. (Two-way ANOVA with Bonferroni post-test). B) ****, p < 0.0001 of differentiated neutrophil : tumor cell co-culture versus tumor cells only from 12 hours to 24 hours and undifferentiated HL-60 Bcl-2 cells : tumor cells co-culture versus differentiated neutrophil : tumor cells co-culture from 9 hours to 24 hours. (Two-way ANOVA with Bonferroni post-test). **C-D.** Graphs of the other two experimental replicates for migration efficiency of undifferentiated HL-60 Bcl-2 cells only, differentiated neutrophils only, MDA-MB-231TD cells only, a 5:1 co-culture of undifferentiated HL-60 Bcl-2 cells and MDA-MB-231TD cells, and a 5:1 co-culture of differentiated neutrophils and MDA-MB-231TD cells towards 200nM fMLP (neutrophil chemoattractant) over the course of 8 hours. Error bars indicate mean ± SD, each experiment was performed in triplicates. C) . ****, p < 0.0001 of tumors cells only versus differentiated neutrophils : tumor cells co-culture from 4 hours to 8 hours and differentiated neutrophils : tumor cells coculture versus undifferentiated HL-60 Bcl-2 cells : tumor cells co-culture from 3.2 hours to 8 hours (Two-way ANOVA with Bonferroni post-test). D) ****, p < 0.0001 of tumors cells only versus differentiated neutrophils : tumor cells co-culture from 4 hours to 8 hours and differentiated neutrophils : tumor cells coculture versus undifferentiated HL-60 Bcl-2 cells : tumor cells co-culture from 1.6 hours to 8 hours (Two-way ANOVA with Bonferroni post-test). **E.** Fluorescent images of an uncoated, formaldehyde-fixed xCelligence migration CIM cartridge with GFP-labeled MDA-MB-231TD tumor cells and mCherry-labeled differentiated neutrophils that migrated through pores towards 10% FBS. Images were taken at 10x magnification using a Nikon Ti2-E inverted microscope. Scale bar = 100µm.

**
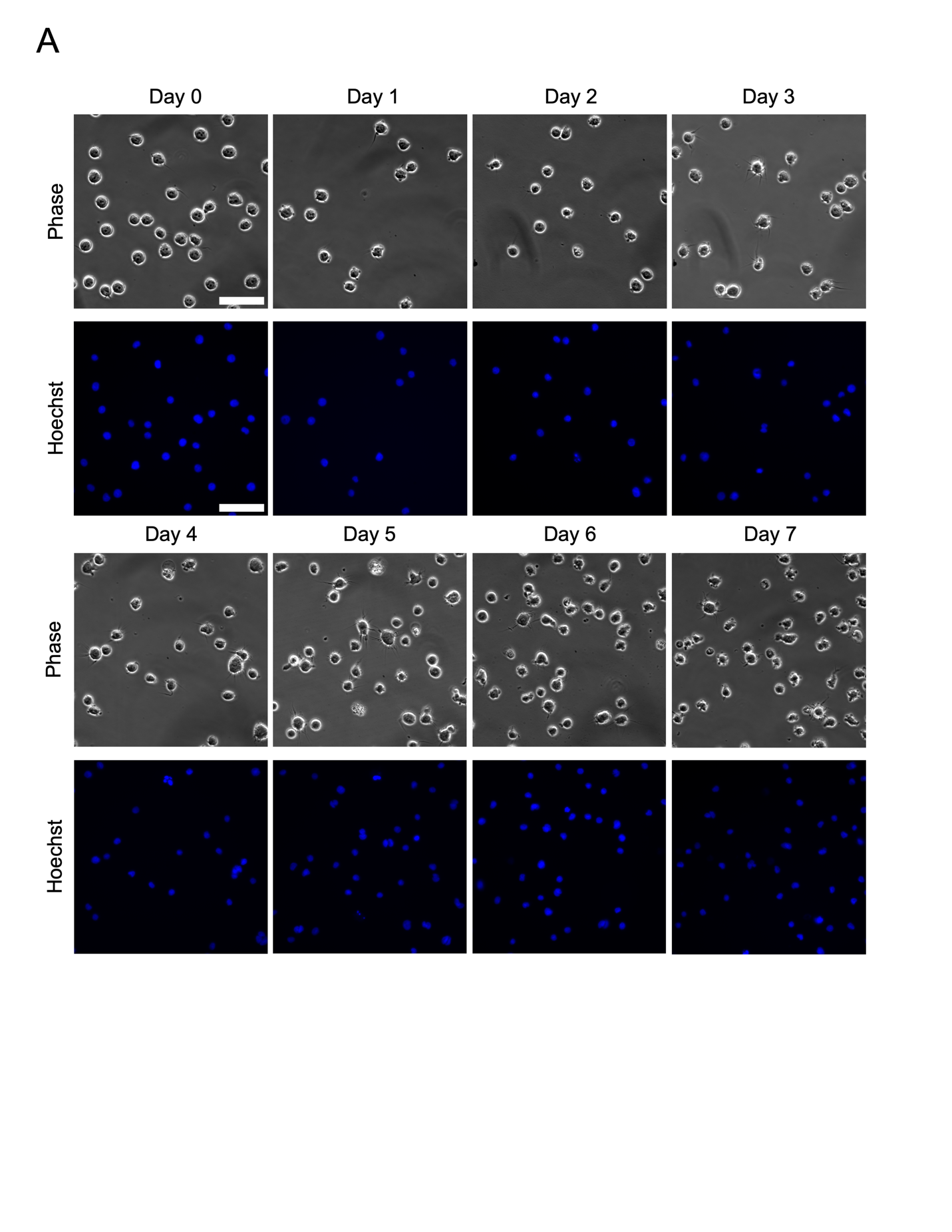
**

**Figure S12: Morphological view of HL-60 Bcl-2 cells differentiation into neutrophils. A.** Representative phase contrast and Hoechst-stained images of tethered, live HL-60 Bcl-2 cells differentiated into neutrophils via 1.3% DMSO over the course of 7 days. Nuclei become more segmented over the course of the differentiation confirming successful neutrophil differentiation. Increased McTNs can also be seen as the differentiation progresses. Scale bar = 50µm.

**Table S1**: Antibodies utilized for western blot and immunofluorescence assays.

| Antibody | Clone | Cat. | Company | Species | WB dilution | IF dilution |
| --- | --- | --- | --- | --- | --- | --- |
| Bcl-2 | 124 | 15071S | CST | Mouse | 1:1000 |  |
| α-tubulin | DM1A | T6199 | Sigma | Mouse | 1:1000 | 1:1000 |
| Detyrosinated α-tubulin |  | ab48389 | Abcam | Rabbit | 1:1000 |  |
| Detyrosinated α-tubulin | RM444 | 31-1335-00 | RevMAb | Rabbit |  | 1:10,000 |
| Acetyl α-tubulin (Lys40) XP | D20G3 | 5335S | CST | Rabbit | 1:1000 | 1:1000 |
| Vimentin | EPR3776 | ab92547 | Abcam | Rabbit | 1:1000 | 1:1000 |
| GAPDH | 6C5 | 32233 | Santa Cruz | Mouse | 1:5000 |  |
| Hoechst 33258 |  | 35569 | Invitrogen |  |  | 1:1000 |

**Table S2**: Antibodies or probes utilized for flow cytometry and surface marker staining.

| Antibody | Clone | Cat. | Company | Fluorescence | Species | Dilution |
| --- | --- | --- | --- | --- | --- | --- |
| Anti-human CD45 | HI30 | 304005 | Biolegend | FITC | Mouse | 1:100 |
| Anti-mouse/human CD11b | M1/70 | 101218 | Biolegend | Alexa Fluor 647 | Rat | 1:100 |
| Anti-human CD11b | ICRF44 | 301310 | Biolegend | APC | Mouse | 1:100 |
| FLPEP |  | F1314 | Life Technologies | FITC |  | 10nM |
| Tubulin Tracker |  | G34075 |  | Oregon Green |  | 1:100 |
| Wheat Germ Agglutinin |  | W11261  W11262 | Invitrogen | Alexa Fluor 488 or 594 |  | 1:100 |
| NucBlue Live ReadyProbes |  | R37605 | Invitrogen | Hoechst |  | Two drops/mL |

**Table S3**: Drugs used throughout the study.

| Chemical | Cat. | Company | Resuspended in | Stock Concentration | Working concentration |
| --- | --- | --- | --- | --- | --- |
| Vinorelbine Tartrate (VR) | 1957-5 | Biovision | PBS | 10mM | 10μM |
| Tetracaine hydrochloride (Tet) | T7508-5g | Sigma | DMSO | 125mM | 250μM |
| Paclitaxel (PTX) | BML-T104-0005 | Enzo | DMSO | 10mM | 1μM |
| Cytochalasin D (CytoD) | C8273-1mg | Sigma | DMSO | 5mM | 5μM |
| Staurosporine | S6942 | Sigma | DMSO | 1mM | 1μM |

**Video S1-4: Neutrophil reattachment with Vinorelbine treatment. S1.** Undifferentiated HL-60 Bcl-2(N) cells do not reattach to fibronectin-coated plates, whereas **S2.** day 7 differentiated neutrophils reattach efficiently to fibronectin. **S3.** PBS-treated neutrophils reattach efficiently to fibronectin-coated plates, whereas **S4.** Vinorelbine treatment causes neutrophils to reattach less efficiently.
